# Ramifications of the HLA-I Allelic Reactivity of Anti-HLA-E*01:01 and Anti-HLA-E*01:03 Heavy Chain Monoclonal Antibodies in Comparison with Anti-HLA-I IgG Reactivity in Non-Alloimmunized Males, Melanoma-Vaccine Recipients, and End-Stage Renal Disease Patients

**DOI:** 10.3390/antib11010018

**Published:** 2022-03-02

**Authors:** Mepur H. Ravindranath, Narendranath M. Ravindranath, Fatiha El Hilali, Senthamil R. Selvan, Edward J. Filippone

**Affiliations:** 1 Department of Hematology and Oncology, Children’s Hospital, Los Angeles, CA 90027, USA; 2 Emeritus Research Scientist at Terasaki Foundation Laboratory, Santa Monica, CA 90064, USA; 3 Norris Dental Science Center, Herman Vostro School of Dentistry, University of Southern California, Los Angeles, CA 90089, USA; nravindr@usc.edu; 4 Faculty of Medicine and Pharmacy of Laayoune, Ibn Zohr University, Agadir 70000, Morocco; f.elhilali@uiz.ac.ma; 5 Tetracore Inc., Rockville, MD 20850, USA; sselvan@tetracore.com; 6 Division of Nephrology, Department of Medicine, Sidney Kimmel Medical College, Thomas Jefferson University, Philadelphia, PA 19145, USA; kidneys@comcast.net

**Keywords:** HLA-Ia, HLA-Ib, HLA-E, heavy chain, open conformers, closed conformers, α1 and α2 helices, β2microglobulin, monomeric, heterodimers, multiplex bead assays, luminex, mean fluorescent intensity (MFI), monospecific, polyreactive, monoclonal IgG antibodies

## Abstract

Serum anti-HLA-I IgG are present in non-alloimmunized males, cancer patients, and transplant recipients. Anti-HLA-I antibodies are also present in intravenous immunoglobulin (IVIg), prepared from the plasma of thousands of healthy donors. However, the HLA-Ia reactivity of IVIg diminishes markedly after passing through HLA-E HC-affinity columns, suggesting that the HLA-I reactivity is due to antibodies formed against HLA-E. Hence, we examined whether anti-HLA-E antibodies can react to HLA-I alleles. Monoclonal IgG antibodies (mAbs) against HCs of two HLA-E alleles were generated in Balb/C mice. The antibodies were analyzed using multiplex bead assays on a Luminex platform for HLA-I reactivity. Beads coated with an array of HLA heterodimers admixed with HCs (LABScreen) were used to examine the binding of IgG to different HLA-Ia (31-HLA-A, 50-HLA-B, and 16-HLA-C) and Ib (2-HLA-E, one each of HLA-F and HLA-G) alleles. A striking diversity in the HLA-Ia and/or HLA-Ib reactivity of mAbs was observed. The number of the mAbs reactive to (1) only HLA-E (*n* = 25); (2) all HLA-Ib isomers (*n* = 8); (3) HLA-E and HLA-B (*n* = 5); (4) HLA-E, HLA-B, and HLA-C (*n* = 30); (5) HLA-E, HLA-A*1101, HLA-B, and HLA-C (*n* = 83); (6) HLA-E, HLA-A, HLA-B, and HLA-C (*n* = 54); and (7) HLA-Ib and HLA-Ia (*n* = 8), in addition to four other minor groups. Monospecificity and polyreactivity were corroborated by HLA-E monospecific and HLA-I shared sequences. The diverse HLA-I reactivity of the mAbs are compared with the pattern of HLA-I reactivity of serum-IgG in non-alloimmunized males, cancer patients, and ESKD patients. The findings unravel the diagnostic potential of the HLA-E monospecific-mAbs and immunomodulatory potentials of IVIg highly mimicking HLA-I polyreactive-mAbs.

## 1. Introduction

HLA class-I expressed on human cell surfaces are heterodimers formed by the non-covalent association of a polymorphic, glycosylated 42-kDa heavy chain (HC) with β2-microglobulin (β2m), a 12-kDa light chain. The HC consists of three helical structures, α1, α2, and α3. HLA-I molecules are polymorphic consisting of several alleles of classical HLA-Ia (HLA-A 6921 alleles, HLA-B 8181 alleles, and HLA-C 6779 alleles) and non-classical HLA-Ib (HLA-E 271 alleles, HLA-F 45 alleles, and HLA-G 88 alleles).

Primarily, intact HLA-I molecules bind to peptides that are derived from proteolytic degradation of cytoplasmic proteins, viruses, and pathogens. The peptide carried in the groove of α1 and α2 domain of the HC interacts with the T cell receptor, and culminates in activation, proliferation, and differentiation of the CD8+ T cells that kill cells that present the specific peptide antigen [1]. After presenting or losing the peptide, HC dissociates from β2m and is released by membrane matrix metalloproteinases (MMPs) [2] from the cell surface into the microenvironment and subsequently the circulation, where it undergoes proteolysis. The HC shedding determines the half-life of HLA-I, which may vary with cells, depending on glycosylation and sialylation [3]. The intact HLA-I molecules are identified with the monoclonal antibody (mAb) W6/32 that recognizes an epitope on β2m (residues 3 and 89) and on the HC residue 121 of all isoforms of HLA-I molecules [4].

In addition, cell surface monomeric (β2m-free HCs) HLA-I variants have been recognized since the 1980s. The HC monomers are found on cells activated by inflammation, infection, trauma, malignancy, cytokines, and chemokines [5,6,7,8,9,10,11,12,13,14,15]. Various functional interactions of these HLA-I monomers have been periodically reviewed [15,16,17,18,19]. The monomeric HLA-I HC functions as a ligand to killer-cell immunoglobulin-like receptors, KIRs (KIE3DS1) on human NK cells and regulates their function [18,19]. These monomers are recognized by the mouse mAb HC-10 but not by mAb W6/32. The epitope of HC-10 is identified between amino acid positions 57 and 62 of the HLA α1 HC; arginine at position 62 (R62) is crucial for recognition [20]. After the shedding of HCs, the amino acid sequences that were masked by β2m are exposed.

The exposure of such cryptic sequences and their fragments is known to elicit an antibody response [21,22,23]. Therefore, one can expect antibodies directed against the HCs of various HLA-I isoforms in normal and healthy individuals. Indeed, natural HLA-Ia Abs occur in normal and healthy human sera and particularly in non-alloimmunized males [24,25,26,27,28,29,30], and they are anticipated to be restricted to native alleles. However, the results of antibody analysis revealed that the antibodies were reactive against several allo-HLA molecules [29,30]. The antibody reactivity of the sera was carried out with LABScreen single antigen bead (SAB) assays using LABScreen beadsets. Examination of the SABs with the mAb W6/32, specific for β2m associated HCs, and the mAb HC-10, confirmed the admixture of HLA-I monomeric HC variants with the intact HLA-I alleles in these beadsets [30,31]. In this context, the primary objective is to understand and elucidate the origin of the allo-HLA reactive Abs occurring in healthy individuals as well as in cancer and transplant patients.

Previously, we have observed that anti-HLA-E mAbs (MEM-E/02, MEM-E/06, MEM-E/07, MEM-E08, and 3D12) not only bind to HLA-E but also to other HLA-I isoforms [32,33], such as the “natural HLA antibodies” observed in the sera of healthy non-alloimmunized males. An inhibition assay with peptides located in the HC, masked by β2m in intact HLA but exposed in β2m-free HCs, was designed to further ascertain the affinity of the mAbs to HLA-E and other HLA-I isoforms. Strikingly, these peptides not only inhibited the mAb binding to HLA-E but also to other HLA-I isomers, namely HLA-A, HLA-B, and HLA-C. These observations suggest that the antibodies reactive to several alleles in normal healthy individuals as well as in those patients waiting for organ transplantation could be those formed against the monomeric variants of HLA found on activated immune cells. If this is true, why are Abs formed against some, but not all, HLA-I alleles of all isomers, although all of them share same sequences such as AYDGKDY?

To address these issues, we have generated monoclonal antibodies to the two recombinant (deglycosylated) heavy chains of HLA-E (HLA-E*01:01 and HLA-E*01:03), which differ in the amino acid at position 107 (HLA-E^R107^ and HLA-E^G107^). Here, we study their reactivity against β2m-free HCs of all HLA-Ia alleles. In doing so, we unravel the striking diversity in the mAbs’ specificity, possibly due to differences in the antigenicity and/or immunogenicity of such epitopes.

## 2. Material and Methods

### 2.1. Production of Murine Monoclonal Antibodies against HCs of HLA-E^R107^ and HLA-E^G107^

Murine monoclonal antibodies were produced following the guidelines approved by the National Research Council’s Committee on Methods of Producing Monoclonal Antibodies [34]. The recombinant polypeptide HCs of HLA-E*01:01 (HLA-E^R107^) and HLA-E*01:03 (HLA-E^G107^) (10 mg/mL in 2-(N-morpholino) ethanesulfonic acid (MES) buffer) were obtained from the Immune Monitoring Laboratory, Fred Hutchinson Cancer Research Center (University of Washington, Seattle, WA, USA). Fifty micrograms of the antigen were diluted in 100 μL of PBS (pH 7.4) and were mixed with 100 μL of the adjuvant TiterMax (Sigma–Aldrich, St Louis, MO, USA) before injection into the footpad and peritoneum of mice. Each antigen was injected to 2 mice. Three immunizations were given at about 12-day intervals, with an additional immunization after 12 days for mice receiving HLA-E^G107^. Only 1 fusion of specific antibody-producing B cells with myeloma tumor cells was carried out for HLA E^G107^. The clones were cultured in a medium containing RPMI 1640 w/L-glutamine and sodium bicarbonate (Sigma–Aldrich, St. Louis, MO, USA, Cat. No. R8758), 15% fetal calf serum, 0.29 mg/mL L-glutamine/Penn-Strept (Gemini-Bio, MedSupply Partners, Atlanta, GA, USA; Cat. No. 400-110), and 1mM sodium pyruvate (Sigma; Cat. No. S8636). Several clones were also grown using Hybridoma Fusion and Cloning Supplement (HFCS; Roche Applied Science, Indianapolis, IN, USA; Cat. No. 11363735001). Isotypes of all the mAbs were characterized, and no IgM Abs were detected.

Almost all the clones were cryopreserved in liquid nitrogen at the Terasaki Research Institute. The hybridomas of monospecific mAbs (TFL-033, TFL-034, TFL-073, TFL-074, and TFL-0145) and polyreactive mAbs (TFL-006 and TFL-007) were patented [35,36], and deposited with American Type Culture Collection, Patent Depository, 10801, University Blvd. (Manassas, VA, USA).

### 2.2. Single Antigen Beads Assay with Single-HLA Antigen-Coated Microbeads

To monitor IgG reactivity to HLA-A, HLA-B, HLA-C, HLA-E, HLA-F, and HLA-G, the culture supernatants as well as human sera, were analyzed with SAB assay using dual-laser flow cytometry on a Luminex platform (Luminex xMAP multiplex technology), as described elsewhere [37]. The beadset consists of a panel of color-coded beads on which individual HLA antigens have been covalently bound (xMap assays) to identify antibody specificities. The LABScreen xMap microbeads contain two reporter fluorophores that are proportionally varied to identify them as one of 100 possible bead identifiers. The array of HLA antigens representing various alleles on the beads are listed on the One Lambda web site (www.onelambda.com (accessed on 4 November 2011)) under Ab-detection products/LABScreen Single Ag Product sheet/HLA-Ia combi-LS1A04-Lot 002 or LS1A04-Lot 005 Worksheet Rev-1. The single recombinant HLA-Ia antigens in LS1A04-lot 007 were used for screening the mAbs. The SAB products in every lot include 31 HLA-A, 50 HLA-B, and 16 HLA-C alleles. The beads supplied by the manufacturer have 2 categories of proteins attached to the beads [32,33,37,38,39]: (1) HLA HC polypeptide only and (2) HC in association with β2m. The mean fluorescent intensity (MFI) values were obtained for IgG antibodies reacting to HLA-coated beads each allele of every HLA isomer. The MFI values were corrected against those obtained with negative control values for each allele.

The recombinant HLA-E, HLA-F, and HLA-G HCs (10 mg/mL in MES buffer) were obtained from the Immune Monitoring Laboratory, Fred Hutchinson Cancer Research Center (University of Washington, Seattle, WA, USA) and were coated specially onto beads by the manufacturers (One Lamda Inc., Los Angeles, CA, USA.) of LABScreen beadset. The HLA-E HCs used for immunization were utilized for coating the beads. The recombinant HLA-E, HLA-F, and HLA-G HCs were individually attached by a process of simple chemical coupling to 5.6-micron polystyrene microspheres, which were internally dyed with infrared fluorophores. Positive beads were coated with murine IgG and negative beads were coated with serum albumin (HSA/BSA). In addition, beads treated with PBS were also used as another negative control. For HLA-E, HLA-F, and HLA-G, the control beads (both positive and negative) were added separately. The culture supernatants were diluted 1:50 in PBS at pH 7.2 and 20-μL were added to the 2-μL of antigen-coated microbeads (at 10 to 1 ratio). Secondary fluorescence-labeled anti-mouse polyclonal Abs were diluted 1/100 (anti-mouse IgG (H + L), Cat. No. 115-116-146, protein concentration at 0.5 mg/mL; Jackson ImmunoResearch Laboratories, West Grove, PA, USA). For identifying the isotypes, anti-Fc secondary mouse anti-isotype Abs (human-absorbed anti-mouse IgG1 (Cat. No. 1070-095) obtained from Southern Biotech, Birmingham, AL, USA) were used at concentration 0.5 mg/mL, after 1:100 dilution.

The reporter fluorophore intensity was measured by a Luminex flow cytometer together with the microbead identifiers, and the fluorescence measurement was classified by bead identifier. The trimmed mean was obtained by trimming a percentage off the high and low ends of a distribution and finding the mean of the remaining distribution. The HLA-Ia microbeads have built-in control beads: positive beads that were coated with murine IgG and negative beads that were coated with serum albumin (HSA/BSA). For HLA-E, HLA-F, and HLA-G, the control beads (both positive and negative) were added separately.

Normalized MFI of mAbs were obtained as follows: ((Trimmed MFI—MFI of mAbs treated with PBS alone)—(negative control beads)). This is an essential step because when antibodies were tested on beads with PBS alone, differential MFI values were observed with different HLA molecules [37,38,39]. Each analysis included data obtained from >100 beads and was performed in duplicate. The MFI cut off used for positive HLA reactivity was 500.

### 2.3. Anti-HLA IgG Purified from the Sera of Non-Alloimmunized Males

The sera used were from non-alloimmunized male volunteers from the Terasaki Foundation Laboratory (TFL). Five milliliters of blood was collected from each volunteer after obtaining informed consent. Fresh sera were used for purification. However, the sera and purified IgG were also frozen and stored at the TFL. The sera were not treated with EDTA. First the sera were tested directly on the SAB assay. To further ascertain the presence of IgG reacting to HLA antigens, IgG was purified from sera using protein-G columns obtained from ThermoFisher Scientific (Rockford, IL, USA). The recombinant protein-G contains two Fc-binding domains and is devoid of albumin and cell surface-binding sites. This was coupled to beaded agarose and ultra-link affinity resins. Protein-G agarose resin binds to all human IgG subclasses but not to IgM, IgD, IgA, or serum albumin. The serum (132 μL) was applied to the protein-G agarose resin after washing the resin twice with PBS, pH 7.2. The serum was mixed well, incubated for 10 min and was passed through the resin. The resin was washed three times with wash buffer (PBS, pH 7.2). The agarose-bound IgG was eluted in three vials using acidic buffer (pH 2.8; 400 μL) and immediately recovered in alkaline buffer (pH 8.5) under centrifugation for a minute, to neutralize the eluates. Either soluble antigen or any other serum factor, such as polypeptides or peptides bound to IgG, would have dissociated at the acid pH used for elution. Three eluates (each 400 μL) were collected and the concentrations of the fractions were determined using the calculations provided in the manual of Eppendorf BioPhotometer (Copyright by 1998 by Eppendorf Netheler—Hinz GmbH, Hamburg, page 87). The calculation of the protein-G fractions was carried out using the calculated factor: C = absorbance at A280—absorbance at A260 to obtain the concentration of the protein (mg/mL). The protein-G eluates (E1, E2, and E3) were tested neat; however, during protein-G elution, the IgGs were diluted 1/10. For this report, we have restricted our observations to data obtained from eluate-2, because the MFIs of E2 were consistently higher than the MFIs of E1 and E3. To detect IgG reactivity to HLA-I alleles in protein G column purified E2 of non-immunized male sera, a multiplex Luminex-based SAB assay was used. The recombinant HLA-Ia antigens in LABScreen beads (1A04-Lot 007) were used for screening. Normalized MFIs of human sera were obtained as follows: ((Trimmed mean MFI—MFI of mAbs treated with PBS alone)—(Negative control bead MFI)—(Negative sera MFI)).

### 2.4. Sera Anti-HLA IgG Antibodies in Melanoma Patients before and after Autologous Vaccine

In this study, we have used sera collected from patients who participated in the clinical trial, NCI-V01-1646, at the Hoag Cancer Center, Newport Beach, CA, USA. The sera and tumor cells were obtained after institutional review board approval and patient consent [40]. The preparation of the autologous vaccine was described in detail elsewhere [40]. Essentially, tumor cell lines were established from the respective biopsies, expanded (to 150 × 10^6^ cells), and treated with IFNγ for 3 days with (1000 U/mL; ACTIMMUNE, InterMune, Brisbane, CA, USA). The treated cells were harvested, irradiated (at 100 Gray) to arrest growth, and cryopreserved until pulsing with autologous dendritic cells (DCs). For final preparation of the vaccine, tumor cells (TCs) obtained from each patient were incubated (overnight at 37 °C) with autologous DCs at a ratio of 1:1 and cryopreserved into aliquots. Just prior to each vaccination, aliquots of DCs loaded with tumor cells were thawed at 37 °C, washed (2× with AIM-V), mixed with GM-CSF (500 μg/mL) with an average TCs-DCs dose of 1.6 × 10^7^ [±0.8 × 10^7^] cells with 78% viability, and were administered to patients subcutaneously. TCs pulsed with DCs, were given weekly for 3 weeks, then monthly for 5 months. Sera were collected on weeks 0 (before immunization), 4, and 24 (after immunization). This study was restricted to six vaccine recipients whose cell lines showed positivity for anti-HLA-E mAb MEM-E/02 (1/1000). The patient characteristics and HLA reactivity of the sera (diluted 1/10) were described elsewhere [41]. To detect the presence of Abs in the sera that react to HLA-E and HLA-Ia alleles, multiplex Luminex^®^-based immunoassay (One Lambda, Canoga Park, CA, USA) was used, as described above.

### 2.5. Sera Anti-HLA IgG Antibodies in HLA-Sensitized End-Stage Kidney Disease Patients Waiting for Transplantation

HLA-I reactivity of the sera of 8 HLA-sensitized end-stage kidney disease (ESKD) patients waiting for donor organs was examined (7 females and 1 male). The causes of ESKD among these patients included diabetes mellitus (DM), hypertensive nephrosclerosis (HN), and lupus erythematosus (LE). Three patients (10DMF73, 12DMF49, and 20HNF64) were possibly sensitized by pregnancy, three (1DMM64, 11DMFDM, and 19HNF45) by transfusion, one (17LEF24) due to prior transplantation, and one (5DM) for unknown reasons. 1DMM64 was transplanted within a year after sample collection for this study. Sera were randomly selected from the Downstate pre-transplant clinic at Sunny Downstate Health Sciences University, University Hospital of Brooklyn (UHB), New York by Prof. Dr. Allen Norin and Dr. Ballabh Das. SAB/DSA study protocols were reviewed and approved by the Downstate Institutional Review Board (IRB), (IRB No. 1232938-3 and No. 341403-1). Sera were examined with LABScreen SABs (Cat. No. 1A04, Lot No. 10). All assays were carried out simultaneously on a single day by one HLA technologist at Terasaki Foundation Laboratory (TFL). For these assays, serum (20 µL) was diluted (1/10) and then incubated with 2 μL of beads for 30 min at room temperature on a shaker. The beads were then washed (3×) with wash buffer. The HLA antigen-IgG binding was assessed with a PE-conjugated secondary-Ab, goat anti—IgH PolyFab (One Lambda Inc., Canoga Park, CA, USA, Cat. No.A82) by incubating the detection-Ab (50 μL at 5 μg/mL) for 30 min at room temperature on a shaker. After washing, the beads were suspended in 1× PBS before acquisition on the Luminex^®^ platform. At least 100 beads were counted for each antigen. The assay includes positive control (coated with human IgG) and negative control (no antigen) beads. We recorded MFI after normalizing the Trimmed mean MFI.

## 3. Results

Heavy chains (HCs) of two different alleles of HLA-E, which differ in their amino acid residue at position 107, were used for immunization. In one allele (HLA-E*01:01, HLA-E^R107^), arginine (R) was found in position 107 and in another (HLA-E*01:03, HLA-E^G107^), arginine was replaced by glycine (G). The monoclonal antibodies generated from these two different alleles were separated as group-R and group-G. Although HCs of each allele were immunized into two mice, only one fusion was carried out for HLA-E^G^. Therefore, the number of mAbs were higher in HLA-E^R^ than in HLA-E^G^.

### 3.1. Classification of Monoclonal Antibodies Formed against HLA-E*01:01 (HLA-E^R^) and HLA-E*01:03 (HLA-E^G^)

Table 1 shows the diverse groups (R-I to R-X and GI to GX) and the number of mAbs developed by immunizing HCs of HLA-E^R^ and HLA-E^G^, and it demonstrates the reactions to different HLA-Ia and HLA-Ib alleles. All mAbs listed in the tables were positive to HLA-E but showed differential reactivity to other alleles, namely 31 HLA-A, 50 HLA-B, 16 HLA-C, and 1 each of HLA-F and HLA-G alleles.

Groups R-I (*n* = 20) and G-I (*n* = 5) are unique in that they are HLA-E monospecific mAbs. They do not recognize (all MFIs <400) any alleles of other HLA-Ia or HLA-Ib alleles.

Groups R-II (*n* = 1) and G-II (*n* = 7) are mAbs specific for HLA-Ib, in that they recognize alleles of HLA-E, HLA-F, and HLA-G but not any of the HLA-Ia alleles.

Group R-III (*n* = 1) is another unique category of HLA-E mAb that recognizes only B*4006 but not any other HLA-Ib or HLA-Ia alleles.

Group R-VI (*n* = 29) and G-VII (*n* = 25) are HLA-E mAbs, that do not recognize any other HLA-Ib loci, such as HLA-F or HLA-G, but recognize all HLA-Ia loci (HLA-A, HLA-B, and HLA-C).

A major category of HLA-E mAbs are those that do not recognize any other HLA-Ib loci but recognize HLA-B, and HLA-C and the A*1101 allele (among all HLA-A alleles) in both R and G groups (R-V: *n* = 68 and G-VI: *n* = 15).

The most interesting R and G groups are those anti-HLA-E mAbs that bind to all other HLA-I loci, namely HLA-A, HLA-B, HLA-C, HLA-F, and HLA-G (R-X: *n* = 2 and G-X: *n* = 4). These are indeed considered as truly HLA-I polyreactive antibodies.

In addition to these antibodies, there are other groups of HLA-E mAbs that bind to different loci in R and G groups. Furthermore, there was one mAb that tested negative for HLA-E but positive only for A*1101 (unpublished).

### 3.2. The Monospecific mAbs Formed against HLA-E*01:01 and HLA-E*01:03

**Figure 1:**
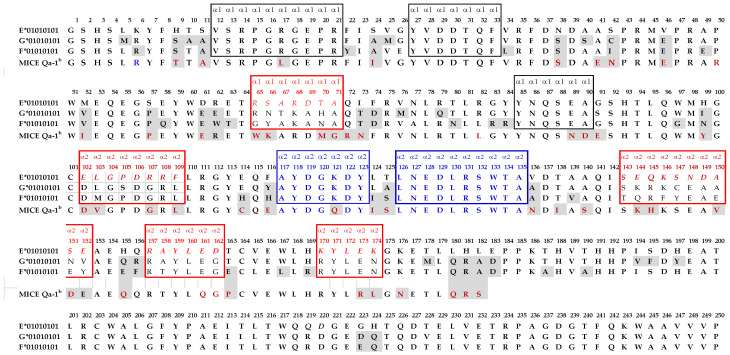
Comparative profile of the amino acid sequences of HLA-E, HLA-G, and HLA-F, and a partial profile of the sequences of mice equivalent of HLA-E, known as Qa1^b^. The amino acid sequences of HLA-E are compared with the amino acid sequences of HLA-G and HLA-F, and also with 504 HLA-A, 844 HLA-B, and 283 HLA-C alleles, to determine the HLA-E specific sequences. The HLA-E specific sequences in α1 and α2 helical domains are shown within the red boxes. HLA-Ib shared epitopes area are shown in black and blue boxes. Sequences in the blue boxes are shared with all HLA-Ia isoforms, while those in black boxes are shared with most, if not all, HLA-Ia alleles.

**Figure 2:**
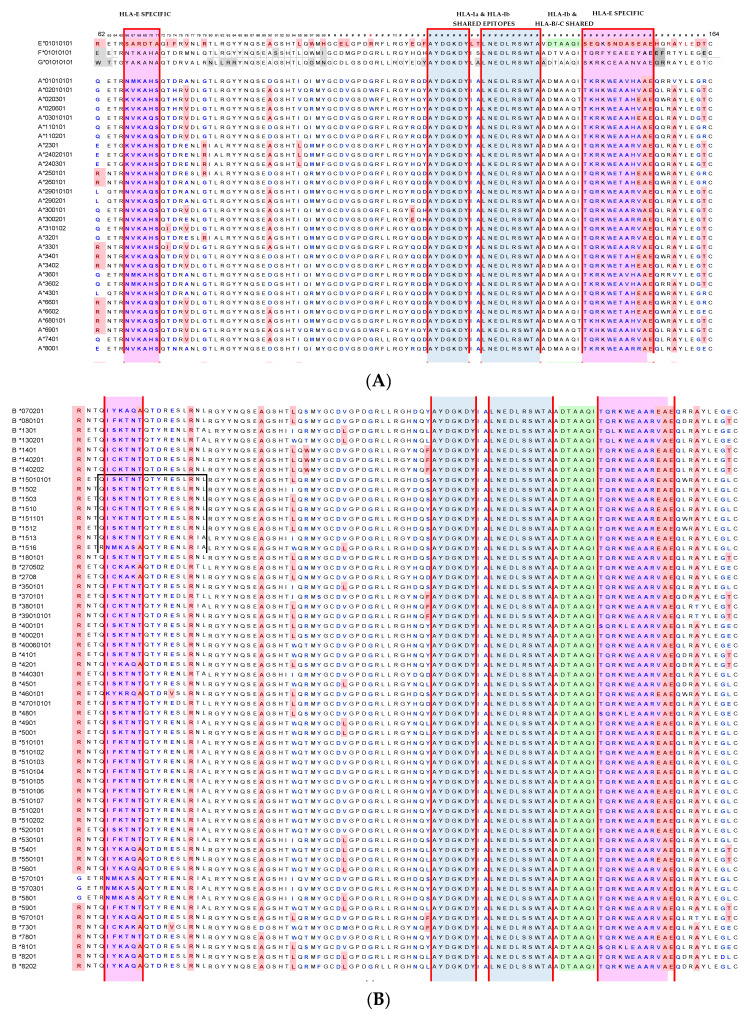

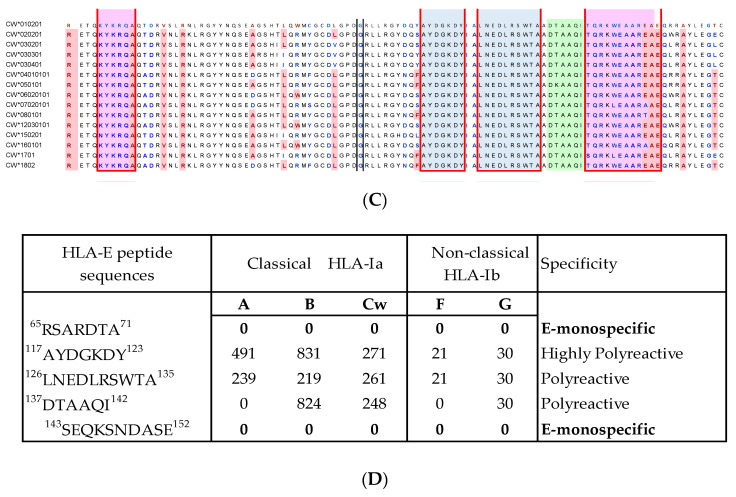
Comparative profile of the amino acid sequences of HLA-Ia alleles (HLA-A, HLA-B, and HLA-C) and HLA-Ib (HLA-E, HLA-F, and HLA-G) isoforms: HLA-Ia and HLA-Ib shared sequences and HLA-Ib and HLA-Band HLA-C shared sequences coated on the microbeads of LABScreen beadsets. (**A**) HLA-E,-F,-G compared with 30 alleles HLA-A coated on the LABScreen beadset; (**B**) 58 alleles HLA-B coated on the LABScreen beadset; (**C**) 15 alleles HLA-C coated on the LABScreen beadset; and (**D**) HLA-E amino acid sequences shared with HLA-Ia and HLA-Ib alleles.

Table 2 illustrates 20 HLA-E monospecific mAbs developed after immunizing two different Balb/c mice with recombinant HLA-E^R107^ and 5 HLA-E monospecific mAbs after immunizing with recombinant HLA-E^G107^. Isotypes of these mAbs were IgG1 in 20/25 mAbs and IgG2a in 3 mAbs out of 25. Isotypes of the two mAbs could not be tested. The MFIs ranged from 10K to 22K for 13 of 25 mAbs. To determine the possible binding sites of these monospecific mAbs, we compared the amino acid sequences of the two HLA-E allelic sequences with the amino acid sequences of 504 HLA-A, 844 HLA-B, and 283 HLA-C alleles. The amino acid sequences of HLA-E, HLA-F, and HLA-G are compared in Figure 1. The HLA-E specific sequences in the α1 and α2 helical domains are shown within the red boxes. Figure 2 provides additional proof for HLA-E monospecificity obtained by comparing the profiles of the amino acid sequences of 30 alleles of HLA-A, 58 alleles of HLA-B, and 15 alleles of HLA-C coated on the microbeads of LABScreen beadsets.

**Figure 3:**
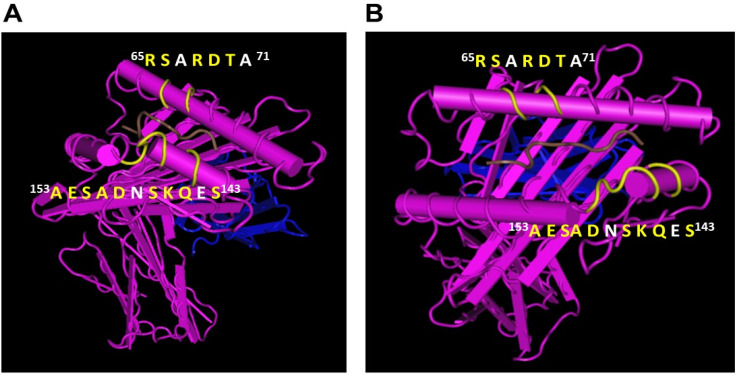
(**A**,**B**) represent the two different conformational orientations of HLA-E specific amino acid sequences exposed on α1 and α2 helices of HLA-E. Note a brown colored peptide sequence in the groove. The monospecific epitopes are on either side of the grove. These sequences are recognized by TFL-033 as evidenced by the binding inhibition of the mAb to the beadsets coated with HLA-E after incubation with the peptide sequences, synthesized and obtained commercially (GenScript Corporation, Piscataway, NJ, USA). It is important to note that the mAb recognizes the sequences, which are not linear but folded and exposing few amino acids (colored yellow) of the epitope for immune recognition; this is an important characteristics of HLA-E mAbs.

To further confirm the monospecificity, we carried out dosimetric inhibition of purified culture supernatants of TFL-033 with two HLA-E-restricted peptides, ^65^RSARDTA^71^ and ^143^SEQKSNDASE^152^, at concentrations ranging from 4.4 to 0.27 mg/well. Although both peptides showed inhibition, the α2 helical peptide SEQKSNDASE showed better dosimetric inhibition than the other peptide [37]. Figure 3 illustrates the conformational orientation of the HLA-E mAb specific epitopes on the α1 and α2 helices of HLA-E. It is important to note that the mAbs do not recognize linear epitopes but recognize conformationally altered epitopes. The yellow letters in the figures represent the exposed amino acids on the epitope, while the amino acids in the white letters represent the masked ones.

Indeed, this is an important characteristic feature of monoclonal antibodies recognizing quaternary structures. The specific amino acids at the center of an epitope that are recognized by an antibody are designated as eplets, and usually consist of one to several amino acids. Another noteworthy finding of the HLA-E sequences is that there are several HLA-E specific sequences in addition to ^65^RSARDTA^71^ and ^143^SEQKSNDASE^152^, as shown in red boxes in Figure 1. These sequences may serve as the epitopes for other HLA-E monospecific mAbs.

### 3.3. The HLA-Ib Specific but HLA-Ia Non-Reactive mAbs

Table 3 documents ten HLA-Ib reactive mAbs that are totally non-reactive to HLA-Ia, of which one (TFL-050) was produced by HLA-E^R^ (Group R-II) and the other nine mAbs were produced by HLA-E^G^. Seven of the nine mAbs were binding to HLA-E, HLA-F and HLA-G (Group G-II). Interestingly the MFI of mAbs reactive to HLA-G are almost equal to or higher than those produced with HLA-E, although the immunogen is HLA-E^G^. Two HLA-E reactive mAbs reacted with either HLA-F (Group G-III) or HLA-G (Group G-IV) at low levels. Figure 1 illustrates some of the amino acid sequences that are common for HLA-Ib isoforms, although these sequences are also found in several but not in all HLA-I alleles. Since we could not decipher specific linear sequences shared among HLA-Ib isoforms, it is inferred that the HLA-Ib specific mAbs may recognize the sequences (in the black boxes) folded in certain unique conformations characteristic for HLA-Ib. Furthermore, the only difference in the amino acid sequences between HLA-E^G107^ and HLA-E^R107^ is the presence glycine in the former, and arginine, at position 107 in the latter. Of the total mAbs obtained after immunizing a single mouse with HLA-E^G107^, nine mAbs are HLA-Ib reactive, whereas only one mAb is reacting to HLA-Ib of the total mAbs obtained after immunizing two mice with HLA-E^R107^, suggesting that the presence of arginine at 107 in HLA-E may impact the conformational orientation of the HLA-E. 

### 3.4. Expanding Diversity of the HLA-Ia Reactivities of HLA-E*01:01 (HLA-E^R^) mAbs

#### 3.4.1. HLA-B*4006 But Not HLA-A, HLA-C, HLA-F, and HLA-G Reactive (Group R-III)

Group R-III represented by mAb TFL-114 in Table 4 illustrates a unique feature of allelic specificity, in that it reacted only with B*4006, in addition to recognizing HLA-E.

#### 3.4.2. HLA-B and HLA-C Reactive But Not HLA-A, HLA-F, or HLA-G Reactive (Group R-IV)

Table 4 also illustrates the evolving diversity of the anti-HLA-E^R^ mAbs represented by the subgroups of Group R-IV. This group is HLA-A, HLA-F, and HLA-G non-reactive. Subgroups A, B, C, D, and E in Table 4 illustrate two important aspects of the mAbs. They are (1) the number of alleles recognized by the mAbs progressively increases from subgroup A to E; and similarly, (2) the MFI of these mAbs for each of the alleles also progressively increases from subgroup A to E. Indeed, 27 out of 28 mAbs (except one) recognize B*4006 positively.

**Figure 4:**
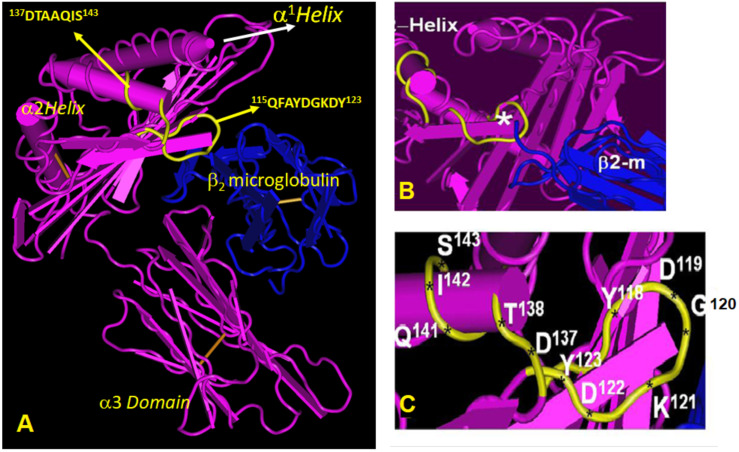
(**A**) The conformational orientation of HLA-E clarifying the location of TFL-006/TFL-007 binding domain (in yellow) in all HLA class I molecule. (**B**) Figure illustrates the steric hinderence caused by β2-microglobulin (blue); therefore ^117^AYDGKDY^123^, is a cryptic epitope not accessible in intact HLA molecules (closed conformers); (**C**) Figure illustrates exposure of ^17^AYDGKDY^123^ on β2-m-free heavy chains of HLA (open conformers).

Figure 2 sheds light on why Group R-IV is not reactive to HLA-A. The figure shows several shared amino acid sequences among HLA-E, HLA-B, and HLA-C (green band). Subgroups C, D, and E may be recognizing ^137^DTAAQI^142^ as evidenced by the alleles carrying this sequence. The sequence ^137^DTAAQI^142^ is found in HLA-E/-G/-B and –C, whereas in HLA-A, it is ^137^DMAAQI^142^ (Figure 2). As shown in Figure 4, ^137^DTAAQI^142^ is evidently a cryptic epitope masked by β2m. Further careful examination of the sequence will reveal that even in the absence of β2M, DTA and DTM are exposed in addition to QI. Therefore, we infer that the R-IV-subgroups, more importantly subgroup E, recognizes ^137^DTAAQI^142^. Peptide inhibition of binding of every one of the R-IV subgroup E mAbs with the beadset may validate the inference.

**Figure 5:**
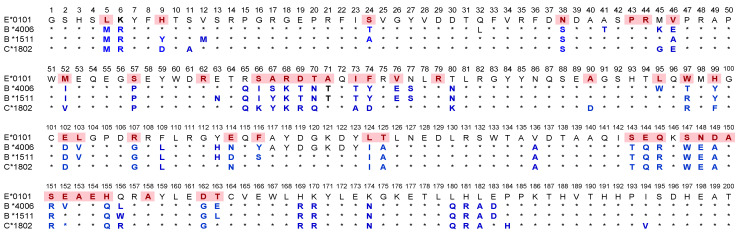
The amino acid sequences of HLA-E compared with the unique B (B*4006, B*1511) or C (C*1802) alleles recognized by a few HLA-E^R^ or HLA-E^G^ mAbs. The asterix in the figure illustrates amino acids, and sequences shared between HLA-E and B*4006 or B*1511 or C*1802.

**Figure 6:**
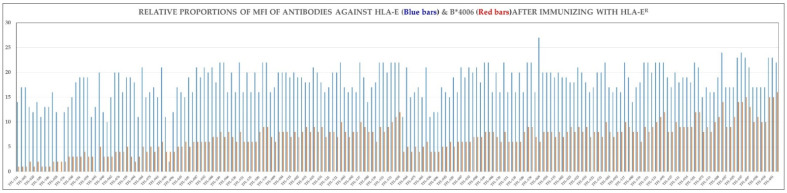
MFI of mAbs reacting to HLA-E^R^ and B*4006, a major HLA-Ia antigen recognized by almost all mAbs.

Since HLA-E mAbs may recognize non-linear sequences, it is possible that the anti-HLA-E mAbs recognize unique conformations of the shared amino acid sequences of B*4006 (Figure 5). In support of this suggestion, Figure 6 illustrates the proportional variations in the MFI of the mAbs reacting to HLA-E and B*4006. The ten mAbs in subgroup A of Group R-IV reveal binding affinity for B*4006 and C*1802. Similarly, six mAbs in subgroup B of the Group R-IV show affinity for C*0501 in addition to recognizing B*4006 and C*1802. The 14 mAbs in subgroups C, D, and E show expanded affinity for other alleles of HLA-B and HLA-C isoforms in addition to the stronger binding affinity for B*4006 and C*1802.

#### 3.4.3. HLA-B and HLA-C Reactive but HLA-F, HLA-G Non-Reactive While Having Unique Affinity for HLA-A*1101 (Group R-V)

The expanding diversity of Group R-IV continues into Group V mAbs, as evidenced by the subgroups A, B, C, D, E, F, and G (Table 5). Group R-V mAbs in general recognized a greater number of alleles of HLA-B and HLA-C compared to Group IV. Most importantly, there was no noteworthy decrease in MFIs of HLA-E reactivity despite the expansion into additional allelic recognition. Subgroups A, B, C, D, E, F, and G in Table 5 also illustrate the two important aspects of the mAbs, namely (1) the number of alleles recognized by the mAbs progressively increases from subgroup A to E, and (2) similarly the MFI of these mAbs for each of the alleles also progressively increased from subgroup A to E. The most striking feature of these subgroups is that they also recognized exclusively HLA-A*1101 but not any other HLA-A alleles. Indeed, all the 68 mAbs recognized B*4006 positively, with a progressive increase in MFI from subgroup A to G.

#### 3.4.4. HLA-Ia Reactive but HLA-F, and HLA-G Non-Reactive (Group R-VI)

The diversity of HLA-E^R^ binding mAbs further expands in Group R-VI, to recognize all of HLA-Ia isoforms (HLA-A, HLA-B, and HLA-C), while not recognizing HLA-F and HLA-G (Table 6). We could identify five subgroups of Group VI. Subgroup A recognized A*3303 in addition to A*1101. In subgroup B, not only the MFIs of mAbs recognizing A*1101 increased, but also there was recognition of A*3601 and A*3401, and much more of A-alleles in other subgroups under Group VI. Group VI in general recognized a greater number of alleles of HLA-B and HLA-C.

Despite such high recognition of HLA-Ia alleles, it is interesting to note the failure to recognize HLA-Ib alleles other than that of HLA-E. Only one allele from each HLA-F and HLA-G was tested, and probably more alleles need to be tested to confirm their lack of affinity of HLA-F and HLA-G. Subgroups A, B, C, D. E, and F in Table 6 also illustrate the two important aspects of the mAbs, namely (1) progressive increase in the number of alleles recognized by the mAbs from subgroup A to E, and similarly, (2) the progressive increase of MFI of these mAbs for each of the alleles also from subgroup A to E. Indeed, all the 29 mAbs recognized B*4006 positively, with a progressive increase in MFI from subgroup A to F.

### 3.5. Expanding Diversity HLA-Ia Reactivity of HLA-E^G^ mAbs

#### 3.5.1. HLA-B Reactive but HLA-A, HLA-C, HLA-F, and HLA-G Non-Reactive (Group G-V)

Four mAbs (TFL-173/174/175/219) represented by Group G-V in Table 7 illustrate a unique feature of allelic specificity not observed earlier among HLA-E^R^ mAbs, that is they reacted only with a few B alleles in addition to HLA-E. Strikingly, TFL-175 recognized B*1511 and B*5201, while the other three mAbs recognized B*5701 in addition. It is important to note that a distinguishing feature of anti-HLA-E^G^ mAbs is that they do not recognize HLA-B*4006, in striking contrast to anti-HLA-E^R^. Figure 5 shows several shared amino acid sequences between HLA-E and B*1511. Since HLA-E mAbs recognize non-linear sequences, it is possible that the anti-HLA-E mAbs recognize unique conformation of the shared amino acid sequences of B*1511, B*5201 and B*5701.

#### 3.5.2. HLA-Ia Reactive but HLA-F, and HLA-G Non-Reactive (Group G-VI)

Group G-VI in Table 7 further illustrates expansion of allelic recognition of HLA-E^G^ mAbs, which included recognition of A*1101 only among alleles of HLA-A isoforms, very similar to Group R-IV of HLA-E^R^ mAbs. It is important to note a progressive increase in the MFIs of Group VI subgroups concomitant with the increase in MFIs of alleles of B and C loci. Such increase in MFI does not affect the MFIs of HLA-E, suggesting the expansion of allelic recognition together with the increase in the mAbs reactivity. No striking increase in MFI was observed for the mAbs that reached saturation for HLA-E recognition, and there was a need to dilute these mAbs further to assess the increase in the mAb strength at the specified concentration. Serial dilution of the mAbs reveal relative affinity of the mAb for different alleles. Group VII in Table 8 illustrates the same, the mAbs in the subgroups are HLA-Ia reactive but HLA-F and HLA-G are non-reactive. Eleven anti-HLA-E^G^ mAbs in Groups B to D recognized B*4006 at low MFI (<2 K).

### 3.6. Emergence of Highly Polyreactive mAbs against HLA-E*01:01 and HLA-E*01:03

#### HLA-Ib Specific mAbs Also Eact with all HLA-Ia Isoform Alleles

HLA-E^R^ as an immunogen has elicited eight HLA-Ib and HLA-Ia reactive antibodies (Table 9). Of these, TFL-069 (R-VII) showed HLA-F reactivity and HLA-A*1101 reactivity in addition to HLA-B and HLA-C reactivities. The MFIs of B*4006 and C*1802 are 7 K and 8 K, respectively. Two mAbs, namely TFL-103 and TFL-104 (R-VIII), failed to react with HLA-F, but reacted well with HLA-G (MFI: 4 K) and with several HLA-A, HLA-B, and HLA-C alleles. Three mAbs showed unique affinity to both HLA-F and HLA-G in addition to HLA-I alleles. Of these mAbs, TFL-049 recognized only HLA-A*1101, four B alleles including B*4006, and three C alleles, including C*1802 (Table 9). The other two mAbs are polyreactive, of which TFL-006 supersedes all anti-HLA mAbs studied so far as being the most highly polyreactive; they recognized 25 A alleles, 45 B alleles, and all the 16 C alleles. Evidently, it recognizes the most common sequences in HLA isoforms located at the α2 helical domain, namely ^117^AYDGKDY^123^ and ^126^LNEDLRSWTA^135^. Indeed, the synthetic peptide AYDGKDY inhibited the binding of TFL-006 and TFL-007 to HLA-I coated beadsets [36]. Figure 4 illustrates the location of the possible epitopes recognized by TFL-006 and TFL-007.

HLA-E^G^ as an immunogen is strikingly different from HLA-E^R^ in eliciting HLA-Ia and Ib reactivity. Ten mAbs (Group G-VIII) showed reactivity only with HLA-F of HLA-Ib isoforms and with almost all alleles of HLA-Ia isoforms. TFL-235 showed remarkably high reactivity with all HLA-A (*n* = 31), HLA-B (*n* = 50), and HLA-C (*n* = 16) alleles tested, whereas the mAb failed to react with HLA-G. On the other hand, Group G-IX, subgroup A (eight mAbs) showed weak reactivity (MFIs 1 K to 2+ K) with HLA-G, but consistently reacted strongly with HLA-A*1101 and weakly with HLA-A*2402, in addition to reacting with several alleles of HLA-B and HLA-C. Group G-IX, subgroup B (three mAbs) reacted strongly with HLA-G (MFIs: 11–14 K) in addition to moderate reactivity with several alleles of HLA-Ia isoforms. The subgroup C (five mAbs) reacted well with HLA-G, but showed very strong reactivity (MFI 8 K–19 K) with several alleles of HLA-B alleles in addition to their reactivity to several HLA-A and HLA-C alleles. Another striking feature of this group is that four of the five mAbs showed MFI of 10 K to 24 K with C*1802.

Group G-X (four mAbs) is unique in that these mAbs reacted with HLA-F and HLA-G in addition to alleles of all the three HLA-Ia isoforms. TFL-232 showed high reactivity to HLA-G (MFI: 21 K). In addition to its reactivity with A*1101 and A*3402, it showed reactivity with HLA-B and HLA-C. The other three mAbs, while showing positivity to HLA-F and HLA-G, reacted reasonably well with alleles of the three HLA-Ia isoforms. The mAb TFL-198 is strikingly different in that it showed weak reactivity with HLA-G (MFI: 0+), moderate reactivity with most of the HLA-A alleles and very strong reactivity with several of HLA-B and HLA-C alleles. None of these three mAbs of G-X met the amazing polyreactivity of R-X mAb TFL-006, specifically to open conformers (Table 10).

### 3.7. HLA-I Reactivity in Human Sera

#### 3.7.1. Non-Alloimmunized Males’ Sera Reactivity to HLA-I Alleles Reflects the Profiles of Some of the HLA-E mAbs

Non-alloimmunized males’ sera showing high positivity for anti-HLA-E IgG antibodies were examined for IgG HLA-I reactivity (Table 11). The following aspects of HLA-I reactivity resemble that of HLA-I reactivity of HLA-E mAbs: (1) HLA-A reactivity of 8 out of 11 sera were low or none (1 to 3 alleles). The highest frequency for HLA-A locus specificities is for A*2402; (2) All sera showed high reactivity for HLA-C; (3) The highest HLA-B frequency was observed for B*4006 = B* 3701 > B*8201; (4) The HLA antibody profile of male donor TJ was strikingly similar to that observed in Group R-IV subgroup B mAbs, presented in Table 4; (5) Similarly, the serum antibody profile of HR was somewhat similar to Group R-IV subgroup E, though HLA-C reactivity was high; and (6) the serum Abs of two male donors (VJ and HO) were similar to the profiles of the mAbs G-VII subgroups C and D.

#### 3.7.2. Pattern of HLA-I Reactivity in Melanoma Patients before and after Immunizing with Autologous IFN-gamma Activated Tumor Cells Reflects HLA-E mAb Profiles

Anti-HLA-E Abs in normal human sera may be responsible for antibody reactivity to allo-HLA-Ia alleles. This provides support to the hypothesis that HLA-E is immunogenic in males. HLA-E and HLA-E HCs are more prevalent on tumor cells, including melanoma, as evidenced by immunostaining with the monospecific mAbs TFL-033 and the polyreactive mAb MEM-E/02, which bind to the cryptic epitopes exposed on the heavy chain of HLA-E [37]. It has been established that IFN-gamma significantly increased the surface expression of HLA-E and the shedding of soluble HLA-E by melanoma tumor cells [42,43]. To assess the immunogenicity of tumor associated HLA-E in cancer patients, specifically in stage III and/or stage IV melanoma patients, sera obtained at weeks 0 and 4 after immunizing with the autologous tumor cells activated by IFN-gamma, were examined for the presence of both HLA-E antibodies and HLA-class Ia allelic reactivity. Table 12 documents that autologous tumor cell vaccination augmented the HLA-E IgG antibodies in the sera and concomitant reactivity against several allo-HLA-Ia alleles. The allo-HLA-Ia reactivity of HLA-E Abs in the vaccine sera was confirmed by inhibiting the HLA-E and HLA-Ia reactivity of the sera with peptide sequences shared between HLA-E and HLA-Ia alleles [41].

#### 3.7.3. Pattern of HLA-I Reactivity of HLA-Sensitized ESKD Patients Waiting for Transplantation

In the end-stage organ disease patients with high levels of preformed anti-HLA antibodies (highly sensitized), transplant rates are significantly reduced because of the increased rejection risk [44]. The highly sensitized patient is destined to remain on the waiting list for extended periods on dialysis, an added risk factor which may decrease both graft and patient survival [45,46]. It is often construed that these antibodies result from exposure to non-self HLA antigens, usually from previous transplants, blood transfusions, and/or pregnancies [47]. Examination of the HLA-reactivity in 10 patients with ESKD, was presented in Table 13, it was noted that the profile of the HLA-I reactivity is similar but not identical to any specific anti-HLA-E mAbs. One patient (11DM) showed reactivity to HLA-A* 1101, and several other A and B alleles, similar to anti-HLA-E mAbs (Groups G-VII E and G). Several patients also showed several similarities in the reactivity of HLA-A and B alleles with anti-HLA-E mAbs (Groups G-VII). These observations suggest that the varying reactivity observed in the patients may be a result of antibody response to HLA-E and possibly to other HLA-Ib alleles with high prevalence of open conformers, such as that of HLA-F and HLA-G.

## 4. Discussion

### 4.1. Immunogenicity of HLA HCs (Open Conformers) versus Intact HLA (Closed Conformers)

A number of investigators [5,6,7,8,9,10,11,12,13,14,15] have demonstrated that HLA-I molecules not only occur as heterodimers on the cell surface, but also occur as monomers or the β2m-free HCs (open conformers). The HLA-I HCs may be involved in functions other than antigen presentation [15,16,17,18,19,48]. The open conformers of HLA alleles expose amino acid sequences, which are cryptic in intact HLA. Therefore, there is a possibility that the immune system may recognize immunogenic domains on the open conformers as “non-self”. Moreover, the cell surface expression of the open conformers is augmented by inflammation, infection, trauma, and malignancy [5,6,7,8,9,10,11,12,13,14,15,19,48]. The breadth and strength of the HLA antibodies, whether directed against HC open conformers or closed conformers, are augmented by the inflammation stimulated by end-stage organ diseases present in transplant patients, as well as by infection, trauma, malignancy, and the transplantation process itself [49,50].

Following peptide presentation or shedding by trimeric closed conformers, the β2m gets separated from the HLA-I HCs. The dissociated HCs, as well as the naturally occurring open conformer HCs are cleaved from the cell surface by calcium-dependent and zinc-containing matrix metalloproteinases (MMPs) [2] and are shed first into the microenvironment and then into the circulation. Upon shedding of HCs, the HC may get deglycosylated and degraded by proteases. Even if we immunize intact HLA in a human or animal model, it will soon be dissociated and degraded into the circulation. Depending on the duration of the degradation, the HCs that can be recognized by the immune cells to elicit antibody responses. The time frame of degradation may vary with the conformational orientation of the amino acid sequences. The partitioning of the diversified groups and subgroups of mAbs against the HCs of HLA-E*0101 and HLA-E*01:03 outlined above supports the above contention.

The exposure of the cryptic sequences and their fragments may elicit an immune response and result in antibody formation against several such epitopes. One has to visualize the HC of a closed conformer and compare it with that of an open conformer. What is common is that the α1 and α2 domains in the immediate vicinity of the peptide groove are exposed in both closed and open conformers (Figure 3). The amino acids between 80 and 140 of the HC are cryptic in closed conformers. No antibody can recognize them in intact HLA (closed conformers) due to the presence of β2m. However, when the HC is devoid of β2m, as in open conformers these sequences are exposed for immune recognition. That explains the ramification of the mAbs reacting to different HLA-I isoforms. One may argue that the diversity of the mAbs is due to immunization of human HLA-E HC molecules in a non-human (mouse) model. Amazingly (as shown in Figure 1), the amino acid sequence of the murine equivalent of HLA-E, namely Qa1^b^, shows similarity to HLA-E comparable to that of HLA-F and HLA-G, particularly in the α1 and α2 domains [51]. There is not much variation in the α3 domain among HLA-I, for this domain is highly shared among all HLA-I isoforms.

### 4.2. HLA-E mAbs Recognizing Private Epitopes

The shed HCs expose two categories of amino acid sequences to be recognized by the immune system. The first category is referred to as “private epitopes”, the sequences specific or unique for an individual isoform or allele. They are the monospecific epitopes. Only the anti-HLA mAbs formed against the monospecific epitopes are reliable for immunodiagnosis of specific HLA-isomers or alleles. Twenty mAbs developed against HLA-E^R^ and five mAbs developed against HLA-E*01:03 (HLA-E^G^) recognized only HLA-E and not HLA-F or HLA-G or any of the isoforms of HLA-Ia. The monospecific or private epitopes are located in the α1 and α2 domains surrounding the peptide groove (Figure 3). To further clarify, it may be stated that the sequences ^65^RSARDTA^71^ and ^143^SEQKSNDASE^152^ are found only in HLA-E alleles (e.g., HLA-E^R^ and HLA-E^G^) and not in any other isoforms (Figure 2 and Figure 3). We have examined more than 500 HLA-A alleles, 800 HLA-B alleles, 280 HLA-C alleles, several HLA-F and HLA-G alleles to ascertain whether ^65^RSARDTA^71^ and ^143^SEQKSNDASE^152^ are indeed monospecific epitopes or “truly private epitopes” specific for all HLA-E alleles or only HLA-E alleles. Most importantly, we have synthesized the sequences ^65^RSARDTA^71^ and ^143^SEQKSNDASE^152^ and tested whether they block the binding of one of the monospecific mAbs (TFL-033) to HLA-E coated beads. The dosimetric inhibition of binding confirms the specificity of the epitopes [37]. The sequence between amino acids 65 and 71 differs markedly as follows: ^65^RSARDTA^71^ (found only in HLA-E); whereas in HLA-F, it is ^65^NTKAHA^71^; in HLA-G, it is ^65^YAKAHA^71^; in all C alleles, it is ^65^KYKRQA^71^; in B*1501, it is ^65^ISKTNT^71^; in B*1510, it is ^65^ICKTNT^71^; and in B*1516, it is ^65^NMKASA^71^. An examination of the amino acid sequences in this region of the peptide groove of both α1 and α2 sequences reveals the diversity of private epitopes.

### 4.3. HLA-E mAbs Recognizing Public Epitopes

The other category of HLA-E epitopes is called “public epitopes”, shared by HLA-E and other HLA-Ia and HLA-Ib isoforms. In a thought-provoking review, Tambur and Class attempted to clarify “that every HLA antigen has its own unique combination of epitopes, but at the same time, many of these epitopes are shared with some other HLA antigens” [52]. However, Figure 2A–C illustrates that “many of these epitopes are not just shared with some other HLA antigens” but shared with almost all HLA-I antigens. Figure 2D points out that ^117^AYDGKDY^123^ is found in 491 HLA-A, 831 HLA-B and 271 HLA-C alleles, ^126^LNEDLRSWTA^135^ in the α2 domain of 239 HLA-A, 219 HLA-B and 261 HLA-C alleles, whereas ^137^DTAAQI^142^ is shared by 824 HLA-B and 248 HLA-C alleles; however, the sequence is altered in HLA-A as ^137^DMAAQI^142^. Indeed, the sequences ^117^AYDGKDY^123^, ^126^LNEDLRSWTA^135^ befit the definition of shared or “public epitopes” (see Figure 1 and Figure 2). In the HLA-I α3-domain, a major fraction of the common amino acid sequences are shared by almost all other HLA-I molecules. The public epitopes in the α1 and α2 domain are always masked by β2-m in intact HLA. The β2-m causes steric hindrance for IgG binding; however, these public epitopes are well exposed in open conformers.

### 4.4. Ramifications of the HLA-I Allelic Reactivity of Anti-HLA-E*01:01 and Anti-HLA-E*01:03 mAbs

**Figure 7:**
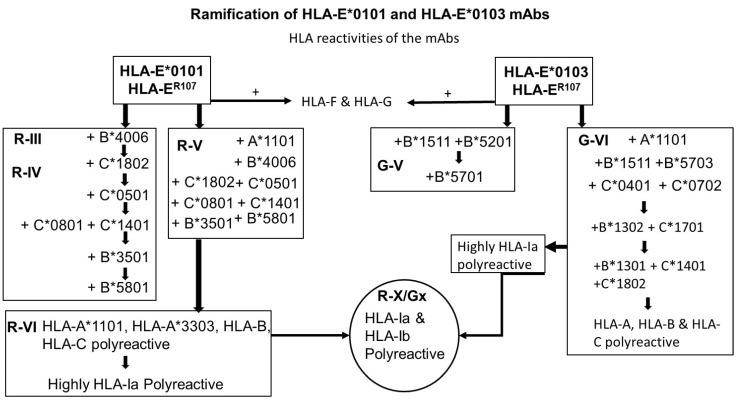
Diagrammatic illustration of the ramification of HLA-I reactivity of the anti-HLA-E*0101 and anti-HLA-E*0103 mAbs.

An interesting finding is that the two HLA-E alleles used for immunization, namely, HLA-E*01:01 (HLA-E^R^) and HLA-E*01:03 (HLA-E^G^) differ by one amino acid substitution at position 107, resulting in an arginine in HLA-E*01:01 (HLA-E^R^) and glycine in HLA-E*01:03 (HLA-E^G^) and there are resulting ramifications on HLA-I allelic reactivity. In contrast to HLA-E^R^, HLA-E^G^ has higher affinity to peptides, higher surface expression, and higher thermal stability, and it is more ancient than HLA-E^R^, though both alleles are presented in human populations in nearly equal frequencies [53]. Interestingly the ramification of the groups of mAbs generated by the two alleles shows some similarities and several striking differences in the pattern of their HLA-I allelic recognition (Figure 7).

A noteworthy similarity of mAbs developed against both alleles is that they recognize HLA-A*1101 exclusively. In fact, we have categorized mAbs negative (R-IV (30 mAbs) and G-V (4 mAbs)) and positive for HLA-A*1101 (Group R-V (68 mAbs) and G-VI (15 mAbs)). Besides one of the HLA-Ib reactive mAb from HLA-E^R^ recognized HLA-A*1101 exclusively.

### 4.5. Many HLA-E mAbs May Recognize Conformationally Altered Sequences

Although all HLA isomers possess both monospecific (private epitopes) and shared (public epitopes) amino acid sequences, the conformational variations of the quaternary structure do not retain all sequences in a linear format. Both Figure 3 and Figure 4 illustrate this point clearly. Consequently, monospecific and polyreactive antibodies do not necessarily recognize linear sequences of the monospecific and shared amino acid sequences. There may be subtle variations introduced by folding due to differences in the other neighboring amino acids. This is based on the following findings:

(a) The only difference in the amino acid sequences of HLA-E^G107^ and HLA-E^R107^ is the presence of glycine in the former, and arginine in the later, at position 107. Interestingly, of the total mAbs obtained after immunizing a single mouse with HLA-E^G107^ or two mice with HLA-E^R107^, eight mAbs of HLA-E^G^ recognized HLA-F, while only one mAb of HLA-E^R^ recognized HLA-F. While eight mAbs of HLA-E^G^ recognized HLA-G, only one mAb of HLA-E^R^ recognized HLA-G. These observations suggest that the presence of glycine at 107 in HLA-E may impact the conformational orientation of the HLA-E to elicit mAbs reactive to HLA-F and HLA-G.
(b) The most striking aspect in their allelic recognition is that almost all mAbs (132 out of 133) developed against HLA-E*01:01 (HLA-E^R^) recognized HLA-B*4006 (Figure 6 and Figure 7). Only 17 anti- HLA-E*01:03 (HLA-E^G^) mAbs showed low reactivity (<4K) to B*4006. Specific recognition of B*4006 by mAbs belonging to groups R-III and R-IV (Table 4) clearly points out the unique exposure of the conformation of B*4006 comparable to HLA-E^R107^, that is not observed with Group G, which is possibly due to the presence of glycine at position 107.
(c) Similarly, HLA-B*1511 is better recognized by the mAbs of HLA-E*01:03 (HLA-E^G^) than those developed from HLA-E^R^. The specific recognition of B*1511 by mAbs belonging to groups G-V and G-VI (Table 7) points out the unique exposure of the conformation of B*5011 is comparable to HLA-E^G107^, in contrast to that of HLA-E^R107^.
(d) HLA-C*1802 was recognized by all mAbs of HLA-E^R^, whereas many mAbs raised against HLA-E^G^ failed to recognize the same allele. Figure 7 diagrammatically illustrates the trends of HLA-I reactivity of the anti-HLA-E*0101 and anti-HLA-E*0103 mAbs.
(e) Group R-IV (Table 4) anti-HLA-E mAbs bind to HLA-B and C but do not bind to any of the HLA-A alleles, possibly due to the presence of ^137^DTAAQI^142^ found in HLA-E/-G/-B and –C), whereas in HLA-A, it is ^137^DTMAAQI^142^ (Figure 2). The T^138^ is replaced by M^138^. Figure 4 clearly shows the location of DTAAQI, in which only DT and QI are exposed during folding. Replacement of “T’ with “M” alters the immune recognition by the 30 mAbs in R-V.
(f) Group R-IV mAbs do not bind to HLA-F, for DTAAQI is modified as ^137^DTVAQI^142^ in HLA-F.
(g) Group R-IV mAbs also do not bind to HLA-G due to change in the closely associated neighboring amino acid of DTAAQI, as follows: in HLA-E, the amino acid preceding DTAAQI is V, whereas in HLA-F and HLA-G, the V is replaced with A (for details see Figure 1).
(h) In ^137^DTAAQI^142^, the amino acid in the 136thposition and in the 143rdposition vary markedly, as seen in Figure 2. These changes may impact the exposure of the sequences. Such specific alterations can impact immune recognition by the mAbs.

### 4.6. Relevance of mAbs to Serum Antibodies of Normal Healthy Humans

Several allo-HLA antibodies are known to occur naturally in the sera of both men and women that have not been alloimmunized by transfusion, prior transplantation, or pregnancy [24,25,26,27,28,29]. Morales–Buenrostro et al. [29] demonstrated the specificities and incidence of the allo-HLA-I Abs in non-alloimmunized males. Neither the origin nor the cause of anti-HLA Abs occurring in healthy individuals have been established. The observations made on the non-alloimmunized sera after purifying with protein-G column, reported in this study, establish the prevalence of anti-HLA-Ia IgG antibodies in the sera of normal, non-alloimmunized individuals. These sera had anti-HLA-E IgG antibodies. It was further observed that the IgG antibodies in the sera recognized several HLA-Ia alleles. The most prevalent alleles recognized by the healthy male sera are B*8201 (*n* = 13), C*0302 (*n* = 13), C*0102 (*n* = 11), B*0702 (*n* = 10), C* 0303 (*n* = 9), B*5601 (*n* = 9), B*4402 (*n* = 8), C*1701 (*n* = 8), B*1502 (*n* = 7), B* 1512 (*n* = 7), C*1502 (*n* = 7), C*1203 (*n* = 7), C*0202 (*n* = 7), B*3701 (*n* = 6), C*0501 (*n* = 6), and C*1802 (*n* = 6). Interestingly most of these sera reacted with HLA-A alleles as observed with anti-HLA-E^R^ and anti-HLA-E^G^ in Table 4 and Table 6.

In an earlier investigation, the presence of HLA-E and HLA-Ia antibodies in normal sera was validated by using commercially available purified human IgG, obtained from Southern Biotek (Birmingham, AL, USA) [28]. These commercial preparations are made from sera of 4 or 5 normal and healthy volunteers, and the purified normal sera IgG are used as a standard for immunoassays. The analysis revealed reactivity of the commercial human IgG to HLA-B (HLA-B*3701) and six other HLA-C alleles (C*0202, C*0501, C*0602, C*0702, C*1601, and C*1802). The alleles of HLA-Ia exhibiting reactivity to the purified human IgG are strikingly similar to HLA-Ia reactivity observed in normal human sera as well as by anti–HLA-E^R^ mAbs in Group R-IV-C (mAb TFL-093). Based on these findings, we infer that most of the anti-HLA antibodies observed in non-alloimmunized males could be autoantibodies generated in the individual against one’s own HLA open conformers, such as that of HLA-E. Of course, there could be a few monospecific mAbs, directed against private or monospecific epitopes. These human HLA-I antibodies are the consequence of shedding of self HLA open conformers (HCs of HLA-A, HLA-B, HLA-C, HLA-E, HLA-F, and HLA-G).

The above inference is also applicable to intravenous immunoglobulin (IVIg), a pharmaceutical preparation of plasma-derived human immunoglobulin G (IgG). It is fractionated from the pooled plasma of 1000 to 60,000 blood donors, including non-alloimmunized donors. Similar to the normal human sera, different IVIg preparations showed a wide variety of reactivity to classic HLA-Ia [54,55,56]. Interestingly, in the previous report, we have ascertained whether the immunoreactivity to HLA-Ia alleles found in IVIg is due to the anti–HLA-E Abs present in IVIg. We have adsorbed out the anti–HLA-E Abs with Sephadex gel conjugated with recombinant HLA-E, then tested for HLA-E and HLA-Ia reactivity. As expected, IVIg immunoreactivity to both HLA-E and HLA-Ia is lost after the adsorbing-out process (see Figure 6A,B in [54]). This previous finding is well supported by the present investigation of anti-HLA-E mAbs.

### 4.7. HLA-E Polyreactive mAbs (TFL-006 and TFL007) Mimic Immunoreactivity of IVIg

Therapeutically, IVIg is administered to reduce allo-HLA antibodies pre- and post-transplantation, but the mechanism of reduction remains unclear. We have compared the efficacy of IVIg and the Group R-X mAb TFL-007, which mimics the HLA-Ia and HLA-Ib reactivity of IVIg in suppressing both antibody production [57] and blastogenesis and proliferation of CD4+ T cells [58]. The mAb-007 significantly suppressed the allo-HLA class-II IgG produced by the B cells, and this suppression was far superior to that by IVIg [55,56]. These findings were confirmed with HLA-I antibody secreted by an immortalized B cell line, developed from the blood of another alloimmunized woman [57]. The binding of the mAb TFL-007 to peptide sequences shared (i.e., shared epitopes) between HLA-Ib and β2m-free HLA HCs (open conformers) on the cell surface of B cells may act as a ligand that induces suppression of IgG production of activated B memory cells.

### 4.8. Profiles of HLA-Ia Reactivity of Anti-HLA-E IgG in Melanoma Patients Reflect the HLA-I Reactivity of Some of the Anti-HLA-E mAbs

Melanoma patients’ sera contain IgG antibodies against HLA-E as well as HLA-B and HLA-C. Interestingly, the HLA-I reactivity in the patients’ sera strongly recalls the pattern observed in Groups R-IV and G-V in that the sera are totally negative for HLA-A alleles. Indeed, the vaccination with autologous tumor cells augmented the MFIs of HLA-E and several other HLA-Ia alleles. However, the pattern of allo-HLA-Ia allelic reactivity of the post-vaccination sera is identical to that observed in pre-immune sera. No new allo-HLA-Ia allelic reactivity was observed in any of the patients after immunization with autologous tumor cell vaccine.

### 4.9. Profiles of HLA-Ia Reactivity of Sensitized Sera of ESKD Patients Reflect the HLA-I Reactivity of the Anti-HLA-E mAbs

The HLA-I reactivity of sensitized patients waiting for donor organs is strikingly different from the normal and melanoma patients’ sera. Sensitized patients showed antibodies for HLA-A*0101, HLA-A*0201, HLA-A*0203, and HLA-A*0301. Among mAbs raised against HLA-E^R^ and HLA-E^G^, two mAbs in Group R-X (TFL-006 and TFL-007), two mAbs in Group G-VII (mAbs TFL-171 and TFL-172), and one in G-GVIII (TFL-235) showed the reactivity to all these alleles. These findings emphasize that the HLA sensitization, as monitored by measuring the strength of HLA antibodies, is a consequence of immune recognition of HLA open conformer on the surface of cells activated by inflammation, infection, and traumas. Furthermore, these findings can be extended to post-transplantation since transplant surgery per se is an inflammation inducer leading to immune recognition of HLA open conformers [49].

## 5. Conclusions

The ramification of HLA-Ia reactivity of the diverse mAbs generated by immunization of the HCs of one HLA-E allele points out the complexity of HLA-I antibodies. This study emphasizes the need to undertake such studies with HLA-F and HLA-G alleles for the purposes of both comparison and elucidation of the patterns of diversity. Correlating the diverse HLA-Ia and HLA-Ib reactivity patterns of serum antibodies after infection, inflammation, autoimmune diseases, and in different cancers and end-stage organ diseases may provide clues to the primary immunogen and the source of the immunogen involved in these diseases. This would be particularly valuable for patients with various cancers and end-stage organ diseases.

Poet William Wordsworth once said that “one impulse from a vernal wood may teach you more of man, of moral evil and of good, than all the sages can.” We conclude from this investigation that “one heavy chain of an HLA exposed to an immune system may reveal the origin and ramifications of human HLA antibodies, their good and bad potentials, than all the transplant and cancer clinicians can envisage”.

## Figures and Tables

**Table 1:** Diverse HLA class-I isoform reactivity of the monoclonal antibodies generated against two alleles of recombinant HLA-E heavy chains.

Immunogen	Groups	Reactivity of Monoclonal Antibodies with HLA-I Alleles						Number of mAbs
		HLA-Ia			HLA-Ib			
		HLA-A	HLA-B	HLA-C	HLA-E	HLA-F	HLA-G	
HLA-E^R107^	R-I	Negative	Negative	Negative	Positive	Negative	Negative	20
	R-II	Negative	Negative	Negative	Positive	Positive	Positive	1
	R-III	Negative	B*4006+	Negative	Positive	Negative	Negative	1
	R-IV	Negative	Positive	Positive	Positive	Negative	Negative	30
	R-V	A*1101+	Positive	Positive	Positive	Negative	Negative	68
	R-VI	Positive	Positive	Positive	Positive	Negative	Negative	29
	R-VII	A*1101+	Positive	Positive	Positive	Positive	Negative	1
	R-VIII	Positive	Positive	Positive	Positive	Negative	Positive	2
	R-IX	A*1101+	Positive	Positive	Positive	Positive	Positive	1
	R-X	Positive	Positive	Positive	Positive	Positive	Positive	2
HLA-E^G107^	G-I	Negative	Negative	Negative	Positive	Negative	Negative	5
	G-II	Negative	Negative	Negative	Positive	Positive	Positive	7
	G-III	Negative	Negative	Negative	Positive	Positive	Negative	1
	G-IV	Negative	Negative	Negative	Positive	Negative	Positive	1
	G-V	Negative	Positive	Negative	Positive	Negative	Negative	4
	G-VI	A*1101+	Positive	Positive	Positive	Negative	Negative	15
	G-VII	Positive	Positive	Positive	Positive	Negative	Negative	25
	G-VIII	Positive	Positive	Positive	Positive	Positive	Negative	10
	G-IX	Positive	Positive	Positive	Positive	Negative	Positive	16
	G-X	Positive	Positive	Positive	Positive	Positive	Positive	4

**Table 2:** HLA-E monospecific mAbs identified with their hybridoma and subclass, produced after immunizing mice with recombinant (non-glycosylated); HLA-E^R107^ and HLA-E^G107^ heavy chains.

Groups	Number of mAbs	mAb Nomenclature	Hybridoma ID	Antigen (Heavy Chain only) Tested on Beads	Subclass	HLA-E Reactivity in MFI (Shorter Version of MFI)	HLA-F Reactivity (MFI)	HLA-G Reactivity (MFI)	HLA-A Reactivity (MFI)	HLA-B Reactivity (MFI)	HLA-C Reactivity (MFI)
R-I	1	TFL-147	FA5746-30F1C8	HLA-E^R^	IgG1	22,829 (22+)	0	0	0	0	0
	2	TFL-148	FA5746-30F1D10	HLA-E^R^	IgG1	16,644 (16+)	0	0	0	0	0
	3	TFL-110	FT5742-14B1G2	HLA-E^R^	NT	16,226 (16)	0	0	0	0	0
	4	TFL-034	FT5742-8C6F6	HLA-E^R^	IgG1	13,272 (13)	0	0	0	0	0
	5	TFL-125	FA5746-14A5E9	HLA-E^R^	IgG1	13,204 (13)	0	0	0	0	0
	6	TFL-033	FT5742-8C6F4	HLA-E^R^	IgG1	13,025 (13)	0	0	0	0	0
	7	TFL-126	FA5746-14A5F12	HLA-E^R^	IgG1	12,397 (12)	0	0	0	0	0
	8	TFL-074	FT5742-20E2E9	HLA-E^R^	IgG1	10,269 (10)	0	0	0	0	0
	9	TFL-073	FT5742-20E2G7	HLA-E^R^	IgG1	10,088 (10)	0	0	0	0	0
	10	TFL-144	FA5746-28D10C6	HLA-E^R^	IgG1	9180 (9)	0	0	0	0	0
	11	TFL-041	FT5742-11B4A2	HLA-E^R^	IgG1	8914 (8+)	0	0	0	0	0
	12	TFL-043	FT5742-11B7F12	HLA-E^R^	IgG1	8448 (8)	0	0	0	0	0
	13	TFL-145	FA5746-29B1C10	HLA-E^R^	IgG1	7622 (7+)	0	0	0	0	0
	14	TFL-042	FT5742-11B4A6	HLA-E^R^	IgG1	7452 (7)	0	0	0	0	0
	15	TFL-182	FA5746-32D10D2	HLA-E^R^	IgG1	7280 (7)	0	0	0	0	0
	16	TFL-001	FT5742-1A1E2	HLA-E^R^	IgG2a	4691 (4+)	0	0	0	0	0
	17	TFL-081	FT5742-23F8C12	HLA-E^R^	IgG1	4261 (4)	0	0	0	0	0
	18	TFL-016	FT5742-4F8F12	HLA-E^R^	IgG2a	1349 (1)	0	0	0	0	0
	19	TFL-047	FT5742-12B4E10	HLA-E^R^	NK	1044 (1)	0	0	0	0	0
	20	TFL-013	FT5742-4B3D8	HLA-E^R^	IgG2a	943 (0+)	0	0	0	0	0
G-I	1	TFL-185	FA5850-1E1E6	HLA-E^G^	IgG1	19,644 (19+)	0	0	0	0	0
	2	TFL-184	FA5850-1E1C2	HLA-E^G^	IgG1	19,227 (19)	0	0	0	0	0
	3	TFL-186	FA5850-1E1C3	HLA-E^G^	IgG1	19,075 (19)	0	0	0	0	0
	4	TFL-226	FA5850-9C3B4	HLA-E^G^ HLA-E^R^	IgG1	18,865 (18+) 18,979 (18+)	0	0	0	0	0
	5	TFL-254	FA5850-14C7F1	HLA-E^G^ HLA-E^R^	IgG1	1604 (1+) 1972 (1+)	0	0	0	0	0

For HLA-A, HLA-B, and HLA-C LABScreen beatsets were tested, which carry both intact HLA (closed conformers) β2m-free heavy chain of HLA (open conformers). For HLA-E, HLA-F, and HLA-G fresh beads coated with β2m-free heavy chains (open conformers) were tested.

**Table 3:** HLA-Ib specific mAbs are non-reactive to HLA-Ia molecules.

Groups	Subgroups	Number of mAbs	mAb Nomenclature	Hybridoma ID	Antigen (Heavy Chain only) Tested on Beads	Subclass	HLA-E Reactivity in MFI	HLA-F Reactivity (MFI)	HLA-G Reactivity (MFI)	HLA-A Reactivity (MFI)	HLA-B Reactivity (MFI)	HLA-C Reactivity (MFI)
R-II		1	TFL-050	FT5742-12B6F3	HLA-E^R^	IgG2b	4876 (4+)	3292 (3)	2683 (2+)	0	0	0
G-II		1	TFL-209	FA5850-5C4F3	HLA-E^G^ HLA-E^R^	IgG1	21,532 (21+) 21,452 (21)	8744 (8+)	20,303 (20)	0	0	0
		2	TFL-208	FA5850-5C4E2	HLA-E^G^ HLA-E^R^	IgG1	21,294 (21) 21,030 (21)	8605 (8+)	20,562 (20+)	0	0	0
		3	TFL-223	FA5850-8D3A4	HLA-E^G^ HLA-E^R^	IgG1	18,447 (18) 21,086 (21)	8587 (8+)	20,587 (20+)	0	0	0
		4	TFL-164	FT5765-1C1B6	HLA-E^G^	IgG2b	15,370 (15)	8230 (8)	24,765 (24+)	0	0	0
		5	TFL-165	FT5765-1C1G5	HLA-E^G^	IgG2b	15,192 (15)	8254 (8)	25,812 (25+)	0	0	0
		6	TFL-162	FT5765-1C1B4	HLA-E^G^	IgG2b	14,667 (14+)	9015 (9)	25,085 (25)	0	0	0
		7	TFL-161	FT5765-1C1B1	HLA-E^G^	IgG2b	14,444 (14)	8607 (8+)	24,714 (24+)	0	0	0
G-III		1	TFL-228	FA5850-9C3B3	HLA-E^G^	IgG1	19,581 (19+) 19,919 (19+)	1134 (1)	0	0	0	0
G-IV		1	TFL-191	FA5850-2C8C10	HLA-E^G^	NK	1000 (1)	0	1016 (1)	0	0	0

Note: HLA-A, HLA-B, and HLA-C LABScreen beatsets were tested, which carry both intact HLA (closed conformers) and (open conformers). NK: not known; for HLA-E, HLA-F, and HLA-G, fresh beads coated with β2m-free heavy chains (open conformers) were tested.

**Table 4:** HLA-E^R^ mAbs: HLA-F, HLA-G, and HLA-A non-reactive but reactive to HLA-B and HLA-C alleles.

MFI Expressed in Thousands																																
Groups	Subgroups	number of mAbs	mAb Nomenclature	Subclass	HLA-E Reactivity (MFI)	HLA-F Reactivity (MFI)	HLA-G Reactivity (MFI)	HLA-A Reactivity (MFI)	HLA-B Reactivity																	HLA-C Reactivity						
									B*0801	B*1301	B*1302	B*1401	B*1501	B*1502	B*1511	B*1512	B*1513	B*1516	B*3501	B*3701	B*4006	B*4601	B*5701	B*5703	B*5801	C*0202	C.*0401	C*0501	C*0602	C*0702	C*0801	C*1802
R-III		1	TFL-114	IgG2a	14+																1											
R-IV	[A]	1	TFL-004	IgG2a	17+																1											1
		2	TFL-005	IgG2a	17																1											1
		3	TFL-019	IgG1	13+																1+											1+
		4	TFL-020	IgG1	12+																1											1+
		5	TFL-099	IgG2a	14																1+											1+
		6	TFL-108	IgG2a	11																1											1
		7	TFL-128	IgG3	13																1											1
		8	TFL-146	IgG1	13																1											1
		9	TFL-084	IgG2a	16																2											1+
		10	TFL-028	IgG1	12+																2											2
	[B]	11	TFL-116	IgG1	8+																1+							1				2
		12	TFL-156	IgG1	12+																2							1				2+
		13	TFL-029	IgG1	13+																2+							1				2+
		14	TFL-030	IgG2a	15																2+							1				2+
		15	TFL-060	IgG2a	18+																2+							1				3
		16	TFL-154	IgG2b	19+																3							1				3
	[C]	17	TFL-158	IgG2b	19							1									4							1+			1	6
		18	TFL-059	IgG2a	19+							1							1		3+							1+				4
		19	TFL-079	IgG1	11+														1		3+				1			1+			1	4
		20	TFL-093	IgG1	13+															1+						1				1		1
		21	TFL-143	IgG2b	20+							1							1		4+				1			1				5
	[D]	22	TFL-090	IgG1	12														1		3				1			2			1	4
		23	TFL-052	IgG2b	10+							1+							1		2+				1			1+			1	3+
		24	TFL-062	IgG1	15							1							1		3+				1			2			1	4
		25	TFL-076	IgG2b	20+							1							1		4				1			1+			1	4+
		26	TFL-076	IgG2b	20+							1							1		4				1			1+			1	4+
		27	TFL-091	IgG1	16+							1							1		4				1			1+			1	5
		28	TFL-159	IgG2b	19+							1+							1		5				1			2	1		1+	7
	[E]	29	TFL-155	IgG2b	19					8+	7+		9	9+	17	4+	15	16+			2+	16+	6+	8								3
		30	TFL-094	IgG2b	18+					12+	8		9	10+	18	5	16+	17+			2	17	6	8								2+

HLA-E^G107^ not tested.

**Table 5:** HLA-E^R^ mAbs: HLA-F and HLA-G non-reactive but reactive to HLA-A*1101, HLA-B, and HLA-C alleles.

MFI expressed in Thousands																																															
Groups	Subgroups	number of mAbs	mAb Nomenclature	Subclass	HLA-E Reactivity (MFI)	HLA-F Reactivity (MFI)	HLA-G Reactivity (MFI)	HLA-A*1101 Reactivity (MFI)	HLA-B Reactivity																												HLA-C Reactivity										
									B*1301	B*1302	B*1401	B*1501	B*1502	B*1511	B*1512	B*1513	B*1516	B*1801	B*3501	B*3701	B*3901	B*4001	B*4002	B*4006	B*4101	B*4403	B*4501	B*4601	B*4701	B*5301	B*5401	B*5701	B*5703	B*5801	B*7801	B*8201	C*0202	C*0303	C*0304	C*0501	C*0602	C*0801	C*1402	C*1502	C*1601	C*1701	C*1802
R-V	[A]	1	TFL-035	IgG1	11			1			1								1					3+										1						2		1+					4+
		2	TFL-064	IgG2b	21			1			1								1					4+										1						1							4
		3	TFL-134	IgG1	15+			1			1+								1					4										1						1+		1					5
		4	TFL-075	IgG1	16			1			1+								1					4+										1						2		1					5
		5	TFL-011	IgG2a	17			1			1								1+					4										1						2		1					1
		6	TFL-061	IgG1	15			1			1+								1+					4+										1+						2+		1+					5
		7	TFL-077	IgG2b	21+			1			1+								1+					6										1+						2+		1					6
	[B]	8	TFL-036	IgG1	11+			1			1								1					4						1				1						2		1+					4+
		9	TFL-078	IgG1	12+			1			1								1					4						1				1						2+		1+					5
		10	TFL-096	IgG1	12+			1			1+								1					3+						1				1						2		1+					4
		11	TFL-012	IgG2a	17+			1			1								1+					4+						1				1+						2		1+					4+
		12	TFL-045	IgG2a	16+			1+			1								1					5						1				1+						2+		1+					5
		13	TFL-160	IgG3	15+			1+			1+								1+					5+						1				1+						2		1					5+
		14	TFL-105	IgG2a	19			1			2								1+					5						1				1+						2+		1+					5+
		15	TFL-120	IgG1	16			1+			2								2					5+						1				1+						2+		1+					6
		16	TFL-087	IgG2b	21+			1+			2								2					6						1				1+						3+		2					7+
	[C]	17	TFL-106	IgG2a	19			1+			2								2					5+		1	1			1				2						3		2					6
		18	TFL-032	IgG2b	21+			1+			2+								2					6		1	1			1				2						3+		2					7
		19	TFL-068	IgG2a	20+			1+			2+								2					6+		1	1			1				2						3+		2					7+
		20	TFL-088	IgG2b	21+			1+			2+								2					7		1	1			1+				2						4		2+					8+
		21	TFL-065	IgG1	18			2			3								2+					7		1	1			1+				2						3+		2					8
		22	TFL-149	IgG2b	22			2+			3								2+				1	8		1	1			1+				2+						3+		2+					4
		23	TFL-150	IgG2b	22+			2			2+								2+				1	7+		1	1			1+				2						3		2					8
		24	TFL-066	IgG1	16+			2			3								2+				1	7+		1	1+			1+				2+						4		2+					8+
		25	TFL-067	IgG2a	20			1+			2+								2				6+	7		1	1			1				2						3+		2					7+
	[D]	26	TFL-130	IgG1	16			1+			2+			1		1			2					5+			1			1				2						2+		1+					6+
		27	TFL-117	IgG2b	22			2			3			1		1			2+				1	8		1	1			1+				2+						3+		2					8+
		28	TFL-151	IgG1	16+			1+			2+								2					5+			1			1				1+						2+	1+	2					6+
		29	TFL-136	IgG2b	20			1			2								1+	1				6			1							1						3	1+	2					8+
		30	TFL-135	IgG1	16			2			3								2				1	6		1	1+			1+				2						3	1+	2+		1	1		7
		31	TFL-152	IgG2b	20			1			2								1+	1				6			1							1						3	2	2		1	1		8+
		32	TFL-100	IgG1	16+			2+			2+							1	3		1		1	7+		1	1+			2				2+						3+		2+					7
		33	TFL-140	IgG2b	22+			2+			3+							1	3				1	8+		1	1+			1+				3						4+		2+					9+
		34	TFL-139	IgG2b	22+			3			3+							1	3+		1		1	9		1	1+			2				3						4+		3					10
		35	TFL-124	IgG1	16			2+			3							1	2+				1	6+	1	1	1+			1+				2+						3+	2	2+		1	1		7+
		36	TFL-009	IgG2a	17+			2			2			1				1	2+		1		1	6	1	1+	1+			1+				2+			1			4		2+					7+
		37	TFL-053	IgG2a	20			2			3							1	2+		1		1	7+	1	1+	1+			1+				2+						4		2+					8
		38	TFL-054	IgG2a	20+			2			3+							1	3		1		1	7+	1	1+	1+			1+				2+						4+		3					8
		39	TFL-089	IgG2a	20+			2			3							1	2+		1		1	7+	1	1+	1+			2				2+						4+		3					8+
		40	TFL-115	IgG2a	19			2			3							1	2+		1		1	7	1	1+	1+			1+				2+						4		2+					8
	[E]	41	TFL-121	IgG1	20+			2+			3		1	1		1		1	3				1	8		1	1+			1+				2+						4		2+					8+
		42	TFL-002	IgG2a	19+			2+			2+		1	1				1	3		1		1	7	1	1+	2			2				3	1					4		2+					7+
		43	TFL-003	IgG2a	19			2+			2+			1				1	3		1		1	7+	1	1+	2			2				3	1					4		3					7+
		44	TFL-021	IgG1	18			3			4		1	1				1	3+		1		1+	8+	1	1+	2			2+	1			3	1					5		3+					9
		45	TFL-022	IgG1	18			3			4		1	1				1	3		1		1	8	1	1+	2			2				3	1					4+		3					8+
		46	TFL-023	IgG2a	21			2+			3+		1	1				1	3		1		1	8+	1	1+	2			2	1			3	1					5		3+					9
		47	TFL-024	IgG2a	20+			2+			4		1	1				1	3		1		1	8	1	1+	2			2				3	1					5		3					0
		48	TFL-031	IgG1	18+			3			3		1	1				1	4		1		1	9	1	1+	2			2+				3+	1					5		3					9+
		49	TFL-123	IgG1	16+			2+			3		1					1	3		1		1	7	1	1+	1+			2				3	1					4		3					8
		50	TFL-122	IgG1	17			3			3+		1					1	3		1		1	7+	1	1+	3			2				3	1					4		3					8+
		51	TFL-131	IgG2a	20+			3			4		1	1		1		1	3+		1	1	1+	7+	1+	2	2			2+	1			3+	1		1			5		3					8
		52	TFL-132	IgG2a	20			2+			3+		1	1				1	3		1		1	6+	1	1+	1+			2	1			3	1					4+		3					7+
	[F]	53	TFL-138	IgG2a	22			3	1		4		1+	2+		2	1+	1	4		1		1+	9+	1	1+	2	1		2+				3+	1					5		3+					10+
		54	TFL-083	IgG1	17+			2+	1+		4		2	4+		3+	3	1	3+		1		1	8	1	1+	2	2+		2				3	1					4+		3					9
		55	TFL-157	IgG1	16			2+	1		3		1+	3+		2+	2	1	3		1		1	7	1	1	1+	1+		2				2+						3+		2+					7+
		56	TFL-092	IgG1	17+			2+	1+		3+		2	4+		3+	2+	1	3	1	1		1	7+	1	1	1+	2		2				2+						4		2+					8
		57	TFL-127	IgG1	16+			3	1+		3+		2	4+		3+	3	1	3+		1		1+	7+	1	1+	2	2+		2	1			3	1					4+		3					8+
		58	TFL-137	IgG2b	22			3+	1		4+		1+	3		2+	2	1	4		1		1+	9+	1	1+	2	1+		2+	1			3+	1					5		3+					10+
		59	TFL-040	IgG2a	19			3+	1		3+		1	1				1+	4		1+		1+	8+	1+	2	2+	1		3	1			4	1	1	1			5		3+					9
		60	TFL-080	IgG1	14			2+			5		1	1+		1		2	3+	1	1+	1	1+	8	1+	2	2+	1	1	2+	1			3	1	1	1	1	1	6		4+	1				10
		61	TFL-026	IgG1	17+			3			4		1	1+		1		1+	3		1+	1	1+	7+	1+	2	2+	1	1	2+	1			3	1		1	1		5		3+	1			1	8
	[G]	62	TFL-119	IgG2a	18+			1+	5+	5	2+	7	6+	12+	3+	11+	9+		2					5+		1	1	9+		1		3+	5+	2						3		2					7
		63	TFL-142	IgG2b	22+			3	9+	8+	4+	10+	11	17+	6	15+	15	1	3+		1		1	9	1	1+	2	13+		2		7	9	3+	1					5		3+					10
		64	TFL-153	IgG2b	22			2+	10	8+	4	10+	11	18	6	16	14+	1	3+		1		1	8	1	1+	1+	14		2		7	9	3	1					5		3					10
		65	TFL-118	IgG2a	20+			3+	10+	10	5	12+	12+	20	8	17+	17+	1+	4		1+		1+	9	1	2	2	16		2+	1	8+	10+	3+	1		1			5+		3+					10+
		66	TFL-133	IgG2a	22			3+	11+	10+	5	12+	13	20+	8	17+	17+	1+	4+		1+	1	1+	10	1+	2	2+	16		3	1	9	10+	4	1		1			6		4					11
		67	TFL-141	IgG2b	22+			3+	11+	11	5+	13	13+	21	8	18+	18	1+	4+		1+	1	1+	10+	1+	2	2+	17		3	1	9	11+	4	1		1			6+		4					12
		68	TFL-095	IgG2b	22+			4+	13	13+	6+	17	16+	24	12	21+	20+	2	5	1	2	1	2	11+	1+	2+	3	21		3+	1	12	13+	5	1+	1	1			7+		5					13

**Table 6:** Evolving HLA-E^R^ mAbs: HLA-F, HLA-G non-reactive but reactive to HLA-A, HLA-B, and HLA-C alleles.

		MFI Expressed in Thousands																												
Groups		R-VI																												
Subgroups		[A]				[B]					[C]							[E]					[F]							
Number of mAbs		1	2	3	4	5	6	7	8	9	10	11	12	13	14	15	16	17	18	19	20	21	22	23	24	25	26	27	28	29
mAb Nomenclature		TFL-015	TFL-027	TFL-039	TFL-111	TFL-010	TFL-014	TFL-082	TFL-102	TFL-101	TFL-072	TFL-086	TFL-113	TFL-085	TFL-008	TFL-038	TFL-057	TFL-098	TFL-025	TFL-112	TFL-037	TFL-071	TFL-097	TFL-048	TFL-058	TFL-017	TFL-018	TFL-070	TFL-051	TFL-069
Subclass		IgG2a	IgG1	IgG2a	IgG2a	IgG1	IgG2a	IgG2a	IgG2b	IgG2b	NT	IgG3	IgG2b	IgG3	IgG1	IgG2b	IgG1	IgG1	IgG1	IgG2b	IgG2b	IgG2b	IgG2a	IgG1	IgG1	IgG1	IgG1	IgG2b	IgG2b	IgG2b
HLA-E Reactivity		19+	17+	20	18+	18+	19+	18+	22	21+	15+	17	16	16	19	24	17	17+	17	23	24	23+	21	17+	17+	17+	17+	23	23+	22+
HLA-F Reactivity																														
HLA-G Reactivity																														
HLA-A Reactivity	A*1101	3+	3+	4	3+	4	4	4	5	5	3	4	3	4+	5+	6+	3+	4+	5+	7	7	8	7	4	4+	4+	4+	8+	8+	9
	A*2402														1	1		1+	2	1+	1	1+	2		1	1	1	2	2	2
	A*2403																	1	1+	1	1	1	1+		1	1	1	1+	1+	1+
	A*2601																							1		1	1			
	A*2901												1	1	1	1	1		1	1+	1	1+	2	1	1+	1+	1+	2	2	2
	A*3001																													1
	A*3002																						1		1	1	1	1+	1+	1+
	A*3201																				1	1	1		1	1	1	1+	1+	1+
	A*3301																1						1	1	1	1	1	1	1+	1+
	A*3303	1	1	1	1	1	1	1	1	1	1	1	1+	1+	1+	1+	1+	1	1+	2	2	2+	3	2	2	2+	2	3+	3+	3+
	A*3401								1	1	1	1	1	1	1	1	1+		1	1+	1+	1+	2	2	2	2	2	2+	2+	3
	A*3601					1	1	1		1		1	1	1	1	1	1	1	1	1+	1+	2	2+	1	1+	1+	1+	2+	2+	3
	A*4301																						1		1	1	1	1	1+	1+
	A*6601																							1						
	A*6802																									1	1			1
	A*8001																											1	1	1
HLA-C Reactivity	C*0102										2+		1				1						1	2	1+	1+	1+	1	1+	1+
	C*0202	1+	1+	1	1	1	1+		1	1	1+	2	2+	2	1+	2+	3	1	2	2+	2+	3	3	4+	3+	3+	3+	4	4	4+
	C*0302										2	1	1+	1	1	1	2		1	1	1	1+	1+	3	2	2+	2	2	2	2+
	C*0303	1	1			1	1	1			1+	1+	2	1+	1+	1+	2+	1	1+	2+	2	2+	3	3+	3	3	3	3+	3+	4
	C*0304		1			1						1+	1+	1+	1	1+	2+	1	1	1+	1+	2	2+	3	3	3	2+	3	3	3+
	C*0401																1							1+	1	1	1			1
	C*0501	5+	5+	5+	5	5+	5+	6	7	7	6+	6	6+	6+	7+	9+	7	6	7+	10	10	10+	9	9+	8	7+	7+	11	11	11+
	C*0602										1		1+	1		1	1+			1	1	1	1+	5	2	2+	2	2	2	2
	C*0702										1		1				1						1	3+	1+	1+	1+	1	1	1+
	C*0801	3+	4	4	3+	4	4	4	5	4+	5	5	4+	5	5+	7	6	4+	6	7+	7+	8	7+	7	7	6	6	9	9+	9+
	C*1203										2						1						1	2	1	1+	1+	1	1	1
	C*1402	1	1	1	1	1	1	1	1	1		1+	2	1+	1+	1+	2+	1	1+	2	2	2	3	4	3	3	3	3+	3+	4
	C*1502										1	1	1+	1	1	1	1+		1	1+	1+	1+	2	2+	2	2+	2	2+	2+	2+
	C*1601	1	1				1+	1			1	1	1+	1		1	1+		1	1	1	1	1+	3	2	2	2	2	2	2
	C*1701		1			1					1+	1	1+	1	1	1	2		1	1	1	1+	2	3+	2+	2+	2+	2+	2+	2+
	C*1802	8+	8+	9+	8+	10	9	9	11+	11+	9+	9+	9+	9+	11+	15	10+	10	11+	14+	15	15+	13	12	12	11	10+	15+	16	15+
HLA-B Reactivity	B*0801																							1+		1				
	B*1301	1	1	1	1	1	1	1	1	1	1	1+	2	1+	1+	3	1+	1+	1+	2+	3+	2+	3+	2	2+	2+	2+	3+	3+	4
	B*1302															1	1				1+		1	1	1	1+	1	1	1+	1+
	B*1401	3+	4+	4	3+	5	3+	4	4+	5	5+	5+	5+	6	6	8+	6	5	6+	8+	9	9	8+	8	8	7	7	10+	10+	11
	B*1402										1	1	1	1		1	1+		1	1	1	1+	2	2+	2	2+	2	2+	2+	2+
	B*1502	1	1+	1	1	1+	1+	1+	1+	1+	2	2	2	2	2	4+	2+	2	2+	3	5	3+	4	3	3	3	3	4+	4+	5
	B*1503												1				1			1	1	1	1+	1+	1+	1+	1+	2	2	2
	B*1510																						1	1	1	1	1	1	1	1
	B*1511	1+	1+	1+	1+	1+	1+	1+	1+	1+	2	2	2+	2	2+	5	2+	2	2+	3+	5+	3+	4	3+	3+	3+	3+	4+	5	5
	B*1512																							1	1	1	1	1	1	1
	B*1513	1	1	1	1	1	1	1	1	1	1+	1+	2	1+	1+	4	2	1	1+	2	4+	2+	2+	2+	2+	2+	2+	3+	3+	3+
	B*1516		1			1	1	1			1	1	1+	1+	1	3	1+	1	1+	2	3+	2	2+	2	2	2+	2	3	3	3
	B*1801	1+	1+	1+	1+	1+	2	2	2	2	2+	2+	3	3	2+	3+	3	2	3	4	4	4	4+	4	3+	4	3+	5	5+	5+
	B*2705																						1	1	1	1	1	1	1	1
	B*2708					1					1	1	1	1		1	1+		1	1	1	1+	2	2	1+	2	2	2	2	2+
	B*3501	4	4	4+	4	4+	4	4+	5+	5+	3+	4+	4	5	5+	7+	4+	5	6	8	8	8+	7+	5+	5+	5+	5	9	9	9+
	B*3701		1			1					2	2	2+	2	1+	0	2+	1	2	2+	3	2+	3+		3	4	3+	4	4	4+
	B*3801																							1		1	1			1
	B*3901	1+	1+	2	1+	2	1+	2	2	2	2	2+	2+	2+	2+	3+	2+	2	3	4	4	4+	4+	3+	3+	3+	3+	5+	4+	6
	B*4001	1	1	‘	1	1	1	1	1	1	1+	1+	2	2	1+	2	2	1+	2	2+	2+	2+	3	2+	3	3	3	3+	3+	4
	B*4002	1+	1+	2	2	2	2	2	2	2+	2	2+	2	2+	2+	3+	2+	2	3	4	4	4	4	3	3+	3+	3	5	5	5+
	B*4006	8	8	9+	8+	9	8+	9	11+	11+	8	9	8	9+	11	14	9	9	11	14	14	14+	12+	10	10+	9+	9+	14+	14+	15+
	B*4101	1+	1+	2	1+	2	2	2	2	2	2+	2+	2+	2+	2+	3	3	2	2+	4	3+	4	4+	3+	3+	3+	3+	5	5	5+
	B*4402					1					1	1	1	1	1	1	1+		1	1+	1	1+	2	2	2	2+	2	2+	2+	2+
	B*4403	2+	2+	2+	2	2+	2+	2+	2+	2+	2	3	2+	3	3+	4	3+	2+	3+	4+	4	5	5	4	4	4	4	6	6	6
	B*4501	2+	3	3	2+	3	3	3	3+	3+	3	4	3+	4	4+	5	4	3+	4+	6+	5+	6+	6	5	5	5	5	7+	7+	8
	B*4601	1	1	1	1	1	1	1	1	1	1+	1+	2	1+	1+	3+	2	1	1+	2+	3+	2+	3	2+	2+	2+	2+	3	3+	3+
	B*4701	1	1	1	1	1	1	1			1+	1+	2	1+	1+	1+	2+	1	1+	2	2	2+	3	3	3	3	3	3+	3+	3+
	B*4801										1	1	1+	1		1	1+			1	1	1	1+	2	2	2	2	2	2	2
	B*5101										1	1	1	1	1	1+	1+		1	1+	1+	1+	2	2	2	2	2	2+	2+	2+
	B*5102					1	1				1	1	1	1	1	1+	1+	1	1+	2	2	2	2+	2	2	2	2	3	3	3+
	B*5201										1	1	1+	1	1	1	1+		1	1+	1+	1+	2	2+	2+	2+	2	2+	2+	2+
	B*5301	2	2+	3	3	3	2	3	4	4	3	3+	3+	4	4+	5+	3+	3+	4+	6+	6	6+	6+	4+	4+	5	4+	7+	7+	8
	B*5401	1+	1+	1+	1+	1+	1+	1+	1+	1+	1+	2	2	2	2	2+	2	2	2+	3+	3	3+	4	3	3	3	3	4+	4+	5
	B*5501										1	1		1	1	1	1		1		1	1+		1+	1+	2	1+	2	2	2+
	B*5801	3+	3+	4	3+	4	4	4	5	5		4	3+	4+	5+	7	4+	4+	5+	7	7+	7+	7+	4+	5	5	5	8+	9	9+
	B*5901											1	1	1		1	1+		1	1	1	1	1+	2	2	2	2	2	2	2+
	B*7301										3		1	1		1	1			1	1	1	1+	1+	1+	1+	1+	2	2	2
	B*7801	1+	1+	1+	1+	1+	1+	1+	2	2	1	2	2	2	2+	3	2	2	2+	3+	3	3+	4	3	3	3	3	4+	5	4
	B*8101										1+															1				
	B*8201	1	1	1	1	1	1	1	1	1	1+	1+	1+	1+	1+	2	2	1	1+	2+	2	2+	3	3	2+	3	2+	3+	3+	3+

Note: For HLA-E only HLA-E^E107^ was tested for MFI.

**Table 7:** HLA-E^G^ mAbs: HLA-F, HLA-G, and HLA-A non-reactive (G-II) or reactive only to HLA-A*1101, and to both HLA-B and HLA-C alleles.

MFI Expressed in Thousands																																										
Groups	Subgroups	number of mAbs	mAb Nomenclature	IgG Subclass	HLA-E^R^ Reactivity (MFI)	HLA-E^G^ Reactivity (MFI)	HLA-F Reactivity (MFI)	HLA-G Reactivity (MFI)	HLA-A*1101 Reactivity (MFI)	HLA-B Reactivity																												HLA-C Reactivity				
										B*0702	B*0801	B*1301	B*1302	B*1402	B*1501	B*1510	B*1511	B*1512	B*1513	B*1516	B*2708	B*3501	B*3901	B*4001	B*4002	B*4201	B*4402	B*4501	B*5001	B*5201	B*5301	B*5401	B*5601	B*5701	B*5703	B*5801	B*8101	C*0401	C*0702	C*1402	C*1701	C*1802
G-V		1	TFL-175	IgG1		12											1+													1+												
		2	TFL-174	IgG1		12											1+													1+				1								
		3	TFL-173	IgG1		12											1+													1+				1								
		4	TFL-219	IgG1	21	20+											2													2				1								
G-VI	[A]	1	TFL-222	IgG1	19	18+			1								3+																		2			1+	1			
		2	TFL-220	IgG1	19	19+			1								3+																		2			1+	1			
		3	TFL-187	IgG2a	25	24+			1+								3+																		2			1+	1			
	[B]	4	TFL-201	IgG1	11	12			1+				1				3																		1+			2+	1+		3	
		5	TFL-193	IgG1	14	15+			2			1	1				5																		2+			3+	2+	1	4	1
		6	TFL-194	IgG1	21	21			2			1	1+				5+																		3			3+	3	1	4+	1
		7	TFL-199	IgG1	15+	15+			2			1	1+				4+																		2+			3+	2+	1	4	1
		8	TFL-258	IgG1	20+	20+			3+			0	1				7						1				1								4+			4	3			1
		9	TFL-257	IgG1	20+	21+			3+			1	1				7						1				1								4			4	3		5	1
	[C]	10	TFL-253	IgG2b	26+	26			6		1	2+	2+		1		11	1		1	1		2		1+	1	2			7+	1	1			7+	1+	1	7	5+	1+	8+	2
		11	TFL-249	IgG2b	26+	25			6		1	2+	2+		1		11	1		1	1		2	2	1+	1	2			7+	1	1	1	1	7+	1+	1	7	5+	1+	9	2
		12	TFL-250	IgG2b	27	26			6		1	2+	2+		1		10+	1					2		1+	1	2			7+	1	1		1	7+	1	1	7	5+	1+	8+	2
		13	TFL-241	IgG1	22	21			4		2+	13+	2	3	8+	1+	7+	14	12				1+		1		1	14	1	5			5+	11	5			4+	4	1	6	3+
		14	TFL-242	IgG1	22	21+			4+		2+	14	2	3+	9	1+	7+	14	12+			1	1+		1		1+	14+	1	5	1		5+	11	5			5	4	1	6	4
		15	TFL-247	IgG1	21+	20+			4		2+	14	2	3	8+	1+	7+	14+	12+				1+		1			14+	1	5	1		5+	11+	5	1		4+		1	6	4

**Table 8:** Evolving HLA-E^G^ mAbs: HLA-F and HLA-G non-reactive but reactive to HLA-A, HLA-B, and HLA-C alleles.

Groups		G-VII																								
		MFI Expressed in Thousands																								
Subgroups		[A]				[B]			[C]					[D]				[E]				[F]			[G]	
Number of mAbs		1	2	3	4	5	6	7	8	9	10	11	12	13	14	15	16	17	18	19	20	21	22	23	24	25
mAb Nomenclature		TFL-221	TFL-218	TFL-217	TFL-216	TFL-196	TFL-167	TFL-231	TFL-252	TFL-170	TFL-169	TFL-166	TFL-168	TFL-251	TFL-207	TFL-206	TFL-205	TFL-243	TFL-246	TFL-244	TFL-245	TFL-189	TFL-190	TFL-188	TFL-172	TFL-171
IgG Subclass		NT	IgG1	IgG1	IgG1	IgG2a	IgG1	IgG2b	IgG2b	IgG1	IgG1	IgG1	IgG1	IgG2b	IgG1	IgG1	IgG1	IgG2a	IgG2a	IgG2a	IgG2a	IgG1	IgG1	IgG1	IgG1	IgG1
HLA-E^R^ Reactivity		25	21	20	20	24		24+	25					26	18+	19		26	24+	25	25	13+	14+	14		
HLA-E^G^ Reactivity		25	20	19+	20	24	15	24	25	17	17+	16+	15	25+	17+	19	18+	25	24+	24+	24+	15+	16+	16	14+	15
HLA-F Reactivity																										
HLA-G Reactivity																										
HLA-A Reactivity	A*0101																								14	15
	A*0201																								1	1
	A*0203																								1+	1+
	A*0206								1	1	1	1	1	1	1	1	1+								1+	1+
	A*0301																								12+	14
	A*1101	6	5	5+	5+	4	5+	8+	9+	6+	6+	6+	6+	9+	8	8	8	12	12	12	12	1+	1+	1+	9+	10
	A*1102																	11	10+	11+	11				11	11+
	A*2402	1	1	1	1	1	1+	2+	3	2	2	2	2	3	3	3	3	2+	2+	3	3				1	1
	A*2403							1	1	1	1	1	1	1	1+	1+	1+									
	A*2601					1	1+	2	1+	2	2	2	2	2+	3	3	3			1	1					
	A*2901						1	1	1+	1+	1+	1+	1+	1+	2	2	2									
	A*3001								1	1	1	1	1	1	1+	1+	1+									
	A*3002						1	1	1	1	1	1	1	1+	2	2	2									
	A*3201						1	1	1	1	1	1	1	1+	1+	1+	1+									
	A*3301							1	1	1	1	1	1	1	1+	1+	1+									
	A*3303						1	1	1+	1+	1+	1+	1+	1+	2	2	2								14+	15
	A*3401																	1	1	1	1				11	12
	A*3402														1	1	1								11+	12
	A*3601								1	1		1	1	1	1+	1+	1+								11	12
	A*4301														1	1	1								11+	12+
	A*7401								1+	1+	1	1	1	1+	1+	1+	1+					1+	2	2+	13+	14
	A*8001																								3	3+
HLA-B Reactivity	B*0702														1	1	1									
	B*0801	15+	3	3	3	1+	2+	23	4	3	3	3	3	4	20+	19	20	1+	1+	1+	1+				11	11+
	B*1301	16+	17	17+	17+	2	4	24	6	4+	4+	4+	4+	6	20+	19+	20	3	3	3	3	<1	1	1	2	2+
	B*1302	3	3	3	3	2	3+	6	6+	4+	4+	4+	4+	7	6	6	6	8	9	8	8+	<1	<1	1	2	2+
	B*1401							1	1+	1	1	1	1	1+	2	2	2	7+	7	7+	8					
	B*1402	4	7+	8	8+			11							12+	12+	12+									
	B*1501	10+	12+	12+	13	1	2	17+	3+	2+	2+	2+	3	*3+*	16+	16+	16+	6	6	6	6+				1	1
	B*1502								1	1	1	1	1	1	1+	1+	1+	15+	15	15+	15+				12	13
	B*1503								1	1				1	1+	1+	1+	10	9+	10	10+					
	B*1510	1+	4	4+	5			5+							8	7+	8								1+	1+
	B*1511	10+	8+	9	8+	6+	8	13+	14	9	9	9	9	14	11+	10+	11	10+	10+	10	10	4	4+	4+	11+	12
	B*1512	17+	17+	17+	18	1	2+	23+	3+	3	3	3	3	3+	20	20	19+	8+	7+	8+	9				1	1
	B*1513	12+	15+	16+	17		1	22	1	1	1	1	1	1	20	18+	19	7+	7	8	8+				14+	15+
	B*1516	1+	1+	1+	1+	1	2	3	3+	2+	2+	2+	2+	3+	3+	3+	3+	1	1+	1+	1+				1	1
	B*1801																								1	1
	B*2705													1	1	1	1									
	B*2708	1	1	1+	1+	1	2	2+	3+	2+	2+	2+	2+	3+	3+	3	3+	1	1	1	1					1
	B*3501	1	1+	1+	1+	1	2	3+	4	3	2+	3	3	4	4	4	4	1+	1	1+	1+				13	14+
	B*3701	0	0	0	0										1	1	1									
	B*3901	2+	2	2+	2+	1+	3	4+	5+	3+	3+	3+	3+	5+	5	5	5	2+	2+	2+	2+				11	12+
	B*4001	1	1	1	1		1+	2	2+	2	2	2	2	2+	3	3	3	1	1	1	1				7	7+
	B*4002	2	1+	2	2	1+	2+	4	5+	3+	3	3+	3+	5	4+	4+	4+	2+	2+	2+	2+				9	9+
	B*4006						1	1	1+	1+	1+	1+	1+	1+	2	2	2									
	B*4201	1+	1	1+	1+	1	2	3	4	2+	2+	2+	2+	*4*	4	4	4	3	2+	3	3				12+	13+
	B*4402	2+	2	2	2	1+	3	4+	5+	3+	3+	3+	3+	5+	5	5	4+	2+	2+	2+	2+				8+	9
	B*4403	0	0	0	0				1	1	1	1	1	1	1+	1+	1+								6	6
	B*4501	16+	18	18+	18+		1+	23+	2	2	1+	2	2	2	20	20	20+								13+	15
	B*4601						1	1	1+	1	1	1	1	1+	2	2	2								10+	11+
	B*4701							1	1	1	1	1	1	1	1+	1+	1+								12	14
	B*4801																	3+	2+	3+	4				9	9+
	B*4901																	4+	4	5	5				8+	9+
	B*5001	1	1+	1+	1+	1	2	3+	4	2+	2+	2+	2+	*4*	4	4	4	6+	5+	6+	7				1	1
	B*5101						1	1	1+	1	1	1	1	1+	2	2	2	5	4	5	5+					
	B*5102							1	1	1	1	1	1	1+	1+	1+	2	3+	3	4	4				11	12
	B*5201	7	6	6+	6+	4+	6+	10+	11	7+	7+	7	7+	11	9+	9+	9+	6+	7	6+	7	2	2+	2+	4+	5
	B*5301	1	1	1+	1+	1	2	2	3+	2+	2+	2+	2+	3+	3	3	3	1	1	1	1+				1	1
	B*5401	1	1	1	1	1	1+	2+	3+	2	2	2	2	3+	3	3	3+	15+	15+	15	15				1	1
	B*5501				9+		1	1	1+	1	1	1	1	1+	1+	1+	1+	0	0	0	0					
	B*5601	7	9	9+	15	1	1+	12+	2+	2+	2	2+	2+	*3*	13	12+	12+	<1	1	1	1				2	2
	B*5701	13	14+	14+	6+	1	2+	20	4	3	3	3	3	*4*	17+	17+	18	1	1	1	1				4+	5
	B*5703	7+	6+	6+	2	4+	6	10+	10+	7	7	7	7	*10+*	9	8	9	7	7	6+	7	2+	3	2+	7	7+
	B*5801	1+	1+	2	1	1	2+	4	4+	3	3	3	3	4+	4+	4+		1+	1+	1+	1+	2+	3	3	1	1+
	B*6701	1	1	1	1+	1	1+	2	2+	2	2	2	2	*2+*	3	3	3		1							
	B*7301						1	1+	2+	1+	1+	1+	1+	2+	2+	2+	2+	12+	12	12	13					
	B*7801					0	0	0	0	0	0	0	0		1	1	1	0	0	0	0					
	B*8101	1	1	1+	0	1	2	3	4	2+	2+	2+	2+	*4*	3+	3+	3+	1+	1+	1+	1+				1	1
	B*8201						1	1	2	1+	1+	1+	1+	2+	2	2	2					12	13	13	14	16
HLA-C Reactivity	C*0102						1		1	1	1	1	1	1+	1+	1+	1+					10+	11+	11+	14	15+
	C*0202						1+	1	2+	1+	1+	1+	1+	2+	2+	2+	2+	13+	11+	13+	13+	13+	13+	14+	13+	15
	C*0302								1	1	1	1	1	1	1+	1+	1+	8	6+	8+	8	16+	16+	16+	14	15+
	C*0303	1	1	1	6	1	2	2	3+	2+	2+	2+	2+	3+	3+	3	3+	9	7+	9	9	15	15	15	14	15+
	C*0304						1	1	2	1+	1+	1+	1+	2	2	2	2					5+	6+	6+	13+	15
	C*0401	6+	5+	6	5+	4+	6	9	10+	7	7	6+	7	10+	8+	8+	8	6+	6+	6+	6+	10	11+	11	13	14
	C*0501						1	1	2	1+	1+	1+	1+	2	2	1+	2	1	1	1	1	20	20	19	13	14+
	C*0602						1	1	1+	1	1	1	1	1+	1+	1+	1+	11+	10	12	12	17+	17+	18	12	14
	C*0702	5+	5+	5+	1				9+	6+	6+	6+	6+	9+	8	7+	7+	5+	5+	5+	5+	11	12+	12+	13+	15
	C*0801								1	1	1	1	1	1	1+	1	1					20	20+	20	15+	17
	C*1203		1	1	2	1	1+	1+	3	2	2	2	2	3	2+	2+	2+	1	1	1	1	13	13+	14	13+	14+
	C*1402	1+	1+	2	7+	1+	2+	3+	4+	3	3	3	3	4+	4	3+	4	19+	19+	19+	19+	18+	18+	18	14+	16
	C*1502						1	1	2	1	1	1	1	2	2	1+	2	24+	24+	24+	24+	18+	19	18+	13+	15
	C*1601					1	1	11	2	1+	1+	1+	1+	2+	2+	2+	2+	22	21+	21+	21+	16	16+	17	13	14+
	C*1701	8+	7	7+	4+	5	7	24+	12	8	8	8	8	12	10	9+	9+	8	8	8	8	11+	12+	12+	14+	16
	C*1802	18	4+	4+	0	1+	3+		5+	4	4	4	4+	6	20+	20	20+	2+	2+	2+	2+	1	1	1	13	14+

**Table 9:** Evolving HLA-E^R^ and HLA-E^G^ mAbs: HLA-Ib mAbs also react to all HLA-Ia isoform alleles.

Groups		R-VII	R-VIII		R-IX	R-X		G-VIII										G-IX																G-X			
		MFI Expressed in Thousands																																			
Subgroups																		[A]								[B]			[C]					[A]		[B]	
Number of mAbs		1	1	2	1	1	2	1	2	3	4	5	6	7	8	9	10	1	2	3	4	5	6	7	8	9	10	11	12	13	14	15	16	1	2	3	4
mAb Nomenclature		TFL-063	TFL-103	TFL-104	TFL-049	TFL-006	TFL-007	TFL-227	TFL-197	TFL-198	TFL-202	TFL-203	TFL-204	TFL-210	TFL-211	TFL-212	TFL-235	TFL-230	TFL-256	TFL-224	TFL-214	TFL-213	TFL-215	TFL-225	TFL-233	TFL-240	TFL-238	TFL-236	TFL-237	TFL-239	TFL-234	TFL-248	TFL-229	TFL-232	TFL-177	TFL-176	TFL-198
IgG Subclass		IgG2b	IgG1	IgG1	IgG2b	IgG2a	IgG2a	IgG1	IgG1	IgG1	IgG1	IgG1	IgG1	IgG1	IgG1	IgG1	IgG1	IgG1	IgG3	IgG1	IgG1	IgG1	IgG1	IgG1	IgG1	IgG1	IgG1	IgG1	IgG1	IgG1	IgG1	IgG1	IgG2b	IgG3	IgG1	IgG1	IgG1
HLA-E^R^ Reactivity		22+	18	17+	15	22+	21+	18	17	16	18	18	17+	19	18	18	21	0	22+	22	20+	21	21	19	19+	18	18	19	20	19+	19	21+	30	22+	14+	13+	15
HLA-E^G^ Reactivity								18+	16	15	17+	16+	16+	17+	17+	18	19+	18+	22	20+	21+	20	20+	19+	18				19+	19+	18	21	27+	21			
HLA-F Reactivity		3+			8+	12+	11	5+	10+	10	11	10+	10+	11	11	11	9																	2+	5	4+	10
HLA-G Reactivity			4+	4+	7+	7+	2+											1	1	1	2	2+	2+	1	1+	11+	14	13+	9+	8	10	2	18+	21+	11	11	0+
HLA-A Reactivity	A*0101					2	1	5+									13										1	1					1	4	0	0	0
	A*0201																3																				
	A*0203					1			2+	2	2+	2+	2+	2	2	2	4+																				2
	A*0206					1			3+	2+	3+	3+	3+	3	3	3	4+																		0	0	2+
	A*0301							3	7	5+	8+	5	5	3+	3	3+	10																				5+
	A*1101	2	6	6	1	10	8	4	11	10	11+	11+	11	11+	11	11	11	4+	4+	4+	5	5	5+	5+	5+	5+	5	5+	7	6+	7	7+	9+		3+	4	10
	A*1102							3	1	1	1+	1	1	1	1	1	9																				1
	A*2301							3+	9+	8	11	10	10+	10	10	10	11																				8
	A*2402		2+	3		3	2	4+	14+	13	15+	14+	14+	14	14	14+	12+	1	1+	1	1	1+	1+	1	1				2+	2+	3	1+	2+		1+	1+	13
	A*2403		2+	2+		3	1+	4+	12+	11	13+	13+	13	13+	12+	13	12												1	1	1+		1		1	1	11
	A*2501					1											4																				
	A*2601					3	1+		1	1	1+	1	1	1	1	1	8									2	2+	2+	2+	2	2+	1+	2		2	2	1
	A*2901		1+	1+		3	2		5	4+	6+	5	5	4+	4	4+	6+												1	1	1	1	1		1	1+	4+
	A*2902					2	1		6+	5	7+	6	5+	4+	4	4+	6																		0	0	5
	A*3001					2	1	5	5	4	6+	4+	4+	4	3+	4	12												1+	1	1+				1	1	4
	A*3002		1	1		3	2	3	7	6	8	6+	6+	6+	6	6+	9+												1	1	1		1				6
	A*3101								4	3	5	3	3+	2+	2	2	3																				3
	A*3201		1	1		2	1+		3+	2+	4+	3	3	2+	2+	2+	5												1	1	1		1			1	2+
	A*3301		1	1		2+	1+		6+	5+	7+	6+	6+	5+	5	5+	5+												1	1	1		1		1	1	5+
	A*3303		2	2		4	2+	1+	7	6+	8	7	7+	7+	7+	7+	8+											1	1	1	1+	1	1		1	1+	6+
	A*3401		1+	1+		6	3+		1+	1	1+	1+	1+	1+	1+	1+	3+																				1
	A*3402					1		4	4+	4	5	4+	5	5	4+	5	11+																	1	0	1	4
	A*3601		2	2		5+	3+		1	1	1	1	1	1	1	1	8+									1	1	1	1		1				1	1	1
	A*4301			1		4	2										5																				
	A*6601					3+	1+		6+	5+	7+	6+	6+	5+	5	5	4+																				5+
	A*6602					1			3+	3	4+	3+	3+	3	3	3	2+																				3
	A*6801					1			5+	4+	6+	5+	5+	5	4	4+	5																				4+
	A*6802		1	1		2	1		2	1+	2	2	2	1+	1+	1+	8																		1+	2	1+
	A*6901					1+			6+	5	7+	5+	5	3+	3+	4	4+																				5
	A*7401							1	2+	2	2+	2+	2+	2+	2	2+	10												1	1	1+		1		1	1	2
	A*8001					2+	1	2	13	13	13+	13	13	13	12+	12+	9																				13
HLA-B Reactivity	B*0702					1		4+	18	17+	18+	18	18	17+	17	18	13+												2	1+	2						17+
	B*0801					2	1	7	2+	2	3	2+	2+	2+	2+	2+	15	1	1	1	1	1	1+	1+	1+	2	2	2	19+	19+	19+	2+	24+	1+	2	2	2
	B*1301		2	2+		5+	3+	8+	18+	19	19_	19	18+	18+	18	18+	16	2	2	2	2+	2+	2+	2+	2+	3+	3	3	19	20+	19+	4+	24	3+	3+	4	18
	B*1302			1		2	1	6	13+	12+	14+	13+	14	13+	13+	13+	13+	2	2	2	2+	2+	2+	2+	3	3	3	3+	6	6	6	3+	6+		2+	2+	12+
	B*1401	2	7	7	2	11	8+	6	15+	14+	16	16	15+	16	16	15+	14												3	2+	3		1+			1	14+
	B*1402		1+	1+		4	2+	2	9+	8	10	10	10+	9+	9	9+	8+												12+	12+	12+		11+				8
	B*1501					1		4	5+	4+	6	5+	6	5+	5+	5+	11+		1	1	1	1	1	1	1	1	1	1	16	16	16	2	18+		1+	2	4+
	B*1502		3	3		6	4	6+	19	18+	19+	19+	19	19	18	18+	15									1	1+	1+	2+	2	2+		5	8	1	1	18+
	B*1503		1	1		2+	1+	6+	16+	16+	17+	17+	17	17	16+	18+	14												1+	1+	1+		1		1	1	16+
	B*1510					2+	1	1	8	7	8+	8	8	7+	7+	7+	6+												8	7+	8		5				7
	B*1511		3	3+		9	5+	9	15	14+	16	15+	15+	15+	15	15+	16+	7+	7+	8	8+	8+	9	9	9	9	9	9+	10+	10	10+	10+	16	1	6	7	14+
	B*1512					1+		7	3+	3	4+	4	4	3+	3	3+	15	1	1	1	1	1	1	1	1	1+	1+	1+	9	19	19+	2	24	2+	2	2+	3
	B*1513		2	2		5	3	6+	1+	1	1+	1+	1+	1+	1+	1+	15												19	19+	18+		22+	4	1	1	1
	B*1516		2	2		5+	3	4+	15	13+	15+	15+	15	15	14+	14+	12	1	1	1	1	1	1	1	1	1	1	2	3	3	3	2+	3		1+	2	13+
	B*1801		3+	3+		6+	4+	2	10+	10	11+	11	10+	10+	10+	10+	7+																				10
	B*2705					2+	1+	1	8+	7+	8+	8+	8+	8	7+	8	6+																				7+
	B*2708		1	1		4	2+	4+	5+	4+	6	5+	5+	5+	5	5+	11+		1		1	1	1	1	1	1	1	1	2+	2	2+	2	2+		2	2	4+
	B*3501	2	6+	6+	1	10	8+	7+	19	19	20	19+	18+	18	18	18	16		1		1	1+	1	1	1	3	3+	3+	8+	8	9	2+	8	9	2	2	19
	B*3701		2+	3		6	4	6	15+	15	16	16	16+	15+	15+	15+	14												1	1	1				1	1	15
	B*3801					3+	1+	3+	13+	12+	14	13+	13+	13	12+	13+	11							2	2	3	3	3	4	4	4	4	5				12+
	B*3901		3+	3+		7	5	7+	19+	19	20+	20	19+	19+	18+	19	15+	1+	2	1+	2	2+	2												2+	3	19
	B*4001		2+	2+		5+	3+	4+	15+	15	16+	16	15+	16	15	15+	12+					1	1			1	1	1	2	1+	2	2	2		1	1	15
	B*4002		3+	3+		6	4+	6	4	3+	4+	4	4+	4	4	4	14	1+	1+	1+	1+	1+	2	2	1+	2	2	2+	3	3	3+	3+	4		2	2+	3+
	B*4006	7	11	11	3	15+	13+		4+	3+	5+	4+	4+	4	3+	4	3+												1	1	1	1	1			1	3+
	B*4101		3+	3+		7	5	3	15+	15	15+	15+	15+	15	15	15	10+																				15
	B*4201							3	9+	8+	11	10	10	9+	9	9	14+	1	1	1	1	1+	1+	1	1	2+	2+	3	3+	3+	3+	2+	9+	12+	2+	3	8+
	B*4402		1+	1+		7	4	1+	6+	6	7+	7	7	6	6	6	10	1+	1+	1+	2	2	2	2	2	2	2	2	3+	3+	3+	3+	4+		2	2+	6
	B*4403		4	4		7	5+	3+			1		1				11																1				
	B*4501	1	5	5+		9+	7+	6+	19	19	20+	19+	19	19	19	19	16									1			19+	21	20	1+	25	2	2	2	19
	B*4601		2+	2+		6	4	6+	17+	17	19	17+	18	17+	17	18	14+												1	1	1+	1	2	1	1	1	17
	B*4701		2	2		6+	3+	8+	20	20	21	19+	19+	19	19	19	16									1+	2	2+	3+	3	3+		2	2	1+	1+	20
	B*4801		1	1		4	2+	7									14+																				
	B*4901							5+									13+																				
	B*5001							8	4	3+	4+	4+	4+	4+	4	4+	15+	1	1	1	1	1	1+	1	1	1+	1+	1+	5	4+	5	2+	3+		2+	2+	3+
	B*5101		1+	1+		6	3+	7+	1+	1+	2	1+	1+	1_	1+	1+	15												2+	2	2+	1	1		1+	1+	1+
	B*5102		2	2		5	3+	7	2	1+	2	2	2	2	2	2	15+												3	2+	3+		1		1	1	1+
	B*5201		1+	1+		4+	2+	9+	10	9	11	10+	10	10	9+	10	15+	5+	5+	5+	6	6	6+	6+	6+	6	6+	6+	8+	9	9	8	12		5+	6+	9
	B*5301	1	5+	5+		8+	7	6+	2+	2+	3	2+	2+	2+	2+	2+	14	1	1	1	1	1	1	1	1	1+	1+	1+	1+	1+	1+	2	2+		1+	2	2+
	B*5401		3	3+		5+	4	5	2+	2+	3	3	3	2+	2+	2+	12+	1	1	1	1	1	1	1	1	1+	1+	1+	3+	3	3+	1	3		1+	1+	2+
	B*5501					2+	1+	2	2+	2	2+	2+	2+	2	2	2	8												1	1	1	1	1				2
	B*5601					1		2+	4+	3+	4+	4+	4+	4	4	4+	10					1	1	1	1	1	1	1	12+	12+	12+	2	13+		1	1	3+
	B*5701							5	5+	4+	6	5+	5+	5+	5+	5+	13	1	1	1	1	1	1+	1	1	1+	1+	1+	17+	18	17+	2+	21+		2	2	4+
	B*5703					1		7	12+	11+	13	12+	12+	12+	12+	12+	13+	5	5+	5+	6	6	6+	6+	6	6+	6	6+	8	8+	8	8	12+	1	5	5+	11+
	B*5801	2	6	6+	1	10	8	9	2+	2	2+	2+	2+	2+	2+	2	17	1	1	1	1+	1+	1+	1+	1+	2+	2+	2+	6	5+	6	2+	5	1	2+	3	2
	B*5901		1	1		5+	3	2	9	8	10	9+	9+	9	9	9	8+																				8
	B*6701							4+	4+	4	5	4+	4+	4+	4+	4+	11												2	2	2	2	2		1	1+	4
	B*7301		1	1		3	2	6+	4	3+	4	4	4	4	3+	4	14									1	1	1	3	3	3	1+	2		1+	1+	3+
	B*7801		3	3+		6	4+	5	18+	17+	19	18+	18+	17+	17+	18	13+												1	1	1						17+
	B*8101					1		7+	19+	19+	20+	19+	19+	18+	19+	18+	16	1		1	1	1+	1+	1	1	2	2+	2+	4+	4+	5	2+	5+	4	2	2	19+
	B*8201		3	2+		4	3	13	20	19	20+	20	19+	19+	19+	19+	18+									1+	1+	1+	1	1	1	1+	2+	3	3	3+	19
HLA-C Reactivity	Cw*0102					7	3	11+	20	29	20+	20+	20	20	19	19+	18+										1	1				1	2	2	2	2+	19
	Cw*0202		2+	2+		10+	6	11	15+	14	16+	15+	15+	11	10+	11	16									1	1	1	1	1	1	1	1+		2	2	14
	Cw*0302		1+	1+		5+	3	10									15+																		1+	2	
	Cw*0303		2	2+		7	4	9+	7+	6+	8+	8	7+	7	6+	7	15+				1	1	1	1	1	1+	1+	1+	1+	1+	1+	2	2+		2+	3	6+
	Cw*0304		2	2		6+	3+	11	2	1+	2+	2+	2+	5+	5	5	17									1	1+	1+				1+	2+	3+	1+	2	1+
	Cw*0401					2+	1	9+	18	18	19+	19+	19	18	18+	18	15+	5	5	5+	5+	6	6	6+	6	6	6	6	6+	6+	6+	7+	12+	2	5+	6	18
	Cw*0501	4	8	8+	2	16	13	13	7	6+	8	7+	7+	12	12	12+	18+									2+	3+	3+	1	1	1	1	5+	13+	2+	2+	6+
	Cw*0602		1	1		9	4	16+	5	4+	6	5+	5	10	9+	10	18+									1+	2	2				1	4+	9+	6	7	4+
	Cw*0702					12	6+	10	19+	17+	19+	19	19+	18+	18+	19	17	4						5	5	5+	5+	5+	6	6	6+	6+	10+	1	5+	6	17+
	CW*0801	3	6	6+	1+	13	10+	11	21+	20	22	21	21	21	21	21	19									1	1+	1+					2+	5	1	1+	20+
	Cw*1203					5	2	12	19+	19+	21	20	19+	19+	19	19	17					1	1			1+	2	2	1+	1	1+	2	3	2+	2+	3	19+
	Cw*1402		2	2		8+	4+	9	10+	9+	11	10+	10	9+	9+	9+	16+							1+	1+	2+	3	3	2	2	2+	3	5	2+	2	2+	9+
	Cw*1502		1+	1+		6	3	11	20+	20+	22	20+	20	20	19+	19+	18+	1	1	1	1+	1+	1+			1+	2	2			1	1+	4	7+	2+	3	20+
	Cw*1601		1	1+		8	4	15+	19+	19+	21	19+	19	18+	19	18+	18					1				1+	1+	1+	1	1	1	2	2+	3+	6	7	19+
	Cw*1701		1+	1+		13+	9	11	13	13	14	13+	14	14	14+	14+	17	6	6	6	7	7	7+	7+	7+	7+	7+	7+	8	8	8	9	15	4	6	6+	13
	Cw*1802	8	11	11+	4	17+	15	8+	9+	8+	10	9+	9+	9	9	9	16+	1+	1+	2	2	2	2+	2	2	3+	3+	3+	19+	20+	10+	4	24+	3	3+	4	6+

**Table 10:** TFL-006 fails to recognize HLA antigens coated on LIFECODES beadsets as they contain only intact HLA (closed conformers).

HLA-I Reactivity with TFL-006 (MFI) at 20 mG/mL *							
HLA-I	LABSCreen		LIFECODES	HLA-I	LABSCreen		LIFECODES
	(Lot # 10)	(Lot # 11)	3005613		(Lot # 10)	(Lot # 11)	3005613
A*01:01	933	852	0	B*07:02	862	670	0
A*02:01	339	542	0	B*08:01	1226	1204	0
A*02:03	1018	156	0	B*13:01	n/a	675	
A*02:06	n/a	616		B*13:02	2514	4923	0
A*03:01	193	210	0	B*14:01	7805	1983	0
A*11:01	4782	3900	0	B*14:02	1831	197	0
A*11:02	537	321	0	B*15:01	335	2541	0
A*23:01	133	125	0	B*15:02	1935	809	0
A*24:02	716	580	0	B*15:03	1822	1146	0
A*24:03	2516	1042	0	B*15:10	n/a	579	
A*25:01	194	349	0	B*15:11	n/a	1658	
A*26:01	2221	2273	0	B*15:12	770	1875	0
A*29:01	1017	1667	0	B*15:13	3135	2437	0
A*29:02	778	715	0	B*15:16	3076	882	0
A*30:01	1496	1243	0	B*18:01	3096	2891	0
A*30:02	n/a	728		B*27:05	634	3252	0
A*31:01	396	687	0	B*27:08	1659	2095	0
A*32:01	515	341	0	B*35:01	6128	517	0
A*33:01	1038	574	0	B*37:01	2650	4352	0
A*33:03	554	1039	0	B*38:01	2521	2425	0
A*34:01	n/a	3002		B*39:01	704	4447	0
A*34:02	1535	1165	0	B*40:01	3429	330	0
A*36:01	1353	941	0	B*40:02	2697	3354	0
A*43:01	2479	2768	0	B*40:06	n/a	1869	
A*66:01	1886	2145	0	B*41:01	3739	1364	0
A*66:02	1454	1327	0	B*42:01	347	957	0
A*68:01	713	454	0	B*44:02	3650	892	0
A*68:02	1185	578	0	B*44:03	1829	2516	0
A*69:01	3128	1786	0	B*45:01	1736	2568	0
A*74:01	652	603	0	B*46:01	3572	1415	0
A*80:01	3132	3758	0	B*47:01	2152	1859	0
				B*48:01	3262	2418	0
				B*49:01	1554	3042	0
			0	B*50:01	1799	4246	0
C*01:02	4066	2820	0	B*51:01	2461	2413	0
C*02:02	7446	3403		B*51:02	n/a	696	
C*03:02	n/a	6614	0	B*52:01	2146	1745	0
C*03:03	2458	3071	0	B*53:01	5442	695	0
C*03:04	4504	8167	0	B*54:01	1662	2427	0
C*04:01	3337	4182	0	B*55:01	2519	4867	0
C*05:01	9124	2567	0	B*56:01	3662	3084	0
C*06:02	5644	5794	0	B*57:01	2089	141	0
C*07:02	8702	1689	0	B*57:03	n/a	1878	
C*08:01	6090	4479		B*58:01	5268	3190	0
C*12:03	n/a	6731	0	B*59:01	3553	1278	0
C*14:02	3937	3627	0	B*67:01	406	3520	0
C*15:02	4465	4651	0	B*73:01	1423	3565	0
C*16:01	4648	2706	0	B*78:01	2996	5271	0
C*17:01	8296	5760	0	B*81:01	1525	2446	0
C*18:02	n/a	829		B*82:02	n/a	1959	

Note: TFL-006 binds to LABScreen beadsets, wherein β2-m-free HLA HCs (open conformers) that are admixed with intact HLA (closed conformers). Alleles in bold are antigens unique to LABScreen, * MFI less than 30 are indicated as 0.

**Table 11:** HLA-Ia reactivity of antibodies of non-alloimmunized males.

HLA Alleles		ID of Non-Alloimmunized Healthy Males										
		Values Expressed as Mean Fluorescent Intensity (MFI)										
		TJ	HR	ME	RC	TP	CM	MR	TS	NR	VJ	HO
HLA-A Reactivity	A*1101										769	898
	A*2301										609	972
	A*2402				616	726	869	632	1099	738	784	1021
	A*2403										557	599
	A*2501									906		
	A*2601									835	613	1094
	A*2901											1013
	A*2902										715	2224
	A*3001										540	
	A*3101											578
	A*3201										539	1511
	A*3301										502	1260
	A*3303											712
	A*3401			958					710	1867	749	2070
	A*3601											
	A*4301									779	562	1660
	A*6601									2535		1387
	A*6602									1830		1382
	A*6802						818				946	
	A*6901								628	618	581	523
	A*7401											716
	A*8001										595	919
HLA-B Reactivity	B*1301				556			564	969	737	722	1025
	B*1302										582	594
	B*1401		524		518	704	506	602	976	830	944	911
	B*1402					552				556	648	624
	B*1502		618								523	573
	B*1503											535
	B*1510										578	1089
	B*1511		592		634	703	667	862	1113	882	1014	1268
	B*1512		562	1923			564		2484			501
	B*1513							507	745	757	713	808
	B*1516				596	572		571	1009	750	1341	2200
	B*3501						727					
	B*3701		748	550	1374	881	1135	725	5103	1203	1215	1901
	B*3801							559				
	B*4001						739					
	B*4006		746		976	932	578	1196	1586	1288	1211	1749
	B*4402		576	2027	546				1375	713	688	779
	B*4403			1777								
	B*4501		673	2044					506			
	B*4601	2821		442	941		741	549		558	871	842
	B*4701											929
	B*4801						697		609		648	537
	B*5101								626	658	687	718
	B*5102								525		656	748
	B*5201								836	565	672	1281
	B*5301								816	693	748	842
	B*5401										565	627
	B*5501								548		541	604
	B*5601								537			1197
	B*5701											
	B*5703										632	829
	B*5801				659	669		653	960	885	934	1193
	B*5901				591	552			658	675	887	900
	B*6701											
	B*7301											583
	B*7801								812	574	680	648
	B*8101						807				543	
	B*8201				799	942		3259	989	1041	994	993
HLA-C Reactivity	C*0102	619	1301	676	1421	1588	1451	1682	1796	1876	1774	2602
	C*0202	680	1314	877	1286	1823	1032	1758	2080	1833	1824	2508
	C*0302	549	1397	759	1226	1280	886	1291	1913	1897	1351	1893
	C*0303		1442	607	937	1073		977	1376	1417	1151	1619
	C*0304	620	1438	689	1211	1395	668	1309	2017	1743	1487	2209
	C*0401	907	1602	1299	2038	2427	1113	2130	2815	2610	2148	4036
	C*0501	628	1149	1078	1268	1406	659	1814	2123	1851	1529	1762
	C*0602	1326	2657	1445	2520	3017	1572	2675	3410	2743	2945	2819
	C*0702	1120	1649	1520	2276	2748	1426	2801	3562	3441	2760	4098
	C*0801		855		926	1027		1117	1423	1274	1281	1514
	C*1203	550	920	672	1187	1402	1207	1430	1631	1502	1590	1820
	C*1402	792	1609	1100	1778	2045	1155	2019	2669	2386	2023	3444
	C*1502		701	509	915	1119	1086	1025	1348	1122	1364	1489
	C*1601	652	1249	1064	1362	1596	877	1510	2044	1731	2038	2093
	C*1701	720	1396	1043	1719	2197	763	1735	2683	2120	1792	1885
	C*1802	1175	1878	1634	2135	2386	1549	2605	3014	2932	2463	2695

Note: Sera were purified using protein-G column and elute-2 alone (1/10 dilution) used for monitoring the HLA-I reactivity using Luminex Single Antigen Bead Assay.

**Table 12:** An unaltered profile of HLA-Ia reactivity in the sera of six melanoma (Stage IV) patients before and 4 weeks after administering autologous melanoma cell vaccine (tumor cells treated with IFNγ were co-cultured with autologous dendritic cells in 1:1 ratio).

Mean Fluorescent Intensity (MFI) of Anti-HLA-Ia IgG Antibodies in Melanoma Patients before and 4 Weeks after Autologus Whole Cell Cancer Vaccine																																									
Patient ID	Before and after 4 Wks Post vaccine	E^R^*0101	HLA-B Reactivity																							HLA-C Reactivity															
			B*0702	B*1301	B*1401	B*1512	B*1516	B*2705	B*2705	B*2708	B*3701	B*4001	B*4002	B*4006	B*4201	B*4402	B*4701	B*5401	B*5501	B*5601	B*5701	B*5703	B*6701	B*8101	B*8201	C*0101	C*0202	C*0302	C*0303	C*0304	C*0401	C*0501	C*0602	C*0702	C*0801	C*1203	C*1402	C*1502	C*1601	C*1701	C*1801
HCC-1763	PreVax	550																																					590		
	PostVax	830																																					1330		
HCC-1766	PreVax	3140																																							
	PostVax	3830								700																															
HCC-1937	PreVax	3710																																							
	PostVax	4040									570																940	650		550			670							1107	
HCC-1896	PreVax	3710	3340					1490	1740			1080	980		1430				1150	820			1210	3250	1170																
	PostVax	4520	3610					1720	1820			990	1030		1480				1190	830			1240	3460	1210																
HCC-2030	PreVax	1650			540	12,330	1180				540			530		8760	540	570			3460	6180	540		6140	750	750	930	500		700	2180	950	1500		660		600		1830	910
	PostVax	8620		640	1230	12,700	2240				790			760		9040	830	950			3190	5520	670		5340	1580	1340	1901	1030	690	1330	2560	1690	2940	650	1240		630		2730	1300
HCC-1957	PreVax	3870			540		640				1250							570									1080	1020			540		770	1150		580				560	
	PostVax	4990			1050		1370				2550							1170									2250	1933	700	1620	880		1370	2040		850				850	

**Table 13:** Profiles of HLA-Ia reactivity of sera of HLA-sensitized ESKD patients waiting for transplantation.

Patient ID		10DM	17LE	12DM	20HN	5DM	19HN	1DM	11DM
No of Alleles	HLA-A	1	9	3	6	10	2	10	22
	HLA-B	11	4	15	14	10	16	12	4
	HLA-C	2	1	1	0	0	2	2	0
HLA-A Reactivity	A*01:01		9470			1694	1255	14,961	3937
	A*02:01		1364			1894		872	
	A*02:03		1034			1809		690	
	A*03:01								5139
	A*11:01								4621
	A*11:02								4519
	A*23:01		1783		1521	13,902		15,118	
	A*24:02		1985		1527	13,190		14,810	
	A*24:03			754	1395	11,895		689	
	A*25:01				1010				4204
	A*26:01								4826
	A*29:02	1634		3543	1090		3014	1596	4622
	A*30:01								4679
	A*31:01								3733
	A*32:01								4744
	A*33:01				1831				4431
	A*33:03								5485
	A*34:02								7463
	A*36:01		2587						5234
	A*43:01		661						3826
	A*66:01								6275
	A*66:02								6318
	A*68:01					1980		537	5555
	A*68:02		823			2054		619	6220
	A*69:01		639			1557			5415
	A*74:01								5083
	A*80:01			537		2209		3355	1010
HLA-B Reactivity	B*07:02	2638		5808			2593		
	B*08:01							1460	
	B*15:12			517	1509				1304
	B*15:16	2057		4253			3698		
	B*27:05				932	1650	591	2757	
	B*27:08				1459	1232			
	B*37:01	808		1495			2849	1095	
	B*38:01	1073		1405			2923	1204	
	B*39:01						917	1559	
	B*40:01	2573		4726			583	549	
	B*40:02		841		710	982	17,106	17,617	
	B*41:01		1288			1142	15,827	17,864	
	B*42:01				1807	3532	776	1469	
	B*44:02	796			549		1373	2117	
	B*44:03	1888		1532			2212		
	B*46:01						613	998	
	B*47:01				1447	3431			
	B*48:01				1612	3014			
	B*49:01				1248	2768			
	B*50:01				1488	1351			
	B*51:01		589	2013					
	B*52:01	1606		3103					
	B*53:01			611	1740				1235
	B*54:01			760	1909				1375
	B*55:01			552	1666				1493
	B*56:01				1260	1578			
	B*57:01	2252		4064			503		
	B*59:01	2527		5685			2546		
	B*67:01	1198	1015	2872			17,248	17,353	
HLA-C	C*03:03		611						
	C*15:02	1283					1630	1300	
	C*17:01	1048		1365			2695	997	

## Data Availability

The original data is available at Terasaki Foundation Laboratory.

## Abbreviations

Abs: antibodies; Ag: antigen; β2m: beta-2 microglobulin; HLA: human leukocyte antigens; HC: heavy chain; mAb: monoclonal antibodies; MFI: mean fluorescent intensity; MMP: matrix metalloproteinases; NK cells: natural killer cells; SAB: single antigen bead.
